# Dominance of highly divergent feline leukemia virus A progeny variants in a cat with recurrent viremia and fatal lymphoma

**DOI:** 10.1186/1742-4690-7-14

**Published:** 2010-02-19

**Authors:** A Katrin Helfer-Hungerbuehler, Valentino Cattori, Felicitas S Boretti, Pete Ossent, Paula Grest, Manfred Reinacher, Manfred Henrich, Eva Bauer, Kim Bauer-Pham, Eva Niederer, Edgar Holznagel, Hans Lutz, Regina Hofmann-Lehmann

**Affiliations:** 1 Clinical Laboratory, Vetsuisse Faculty, University of Zurich, Zurich, Switzerland; 2 Clinic for Small Animal Internal Medicine, Vetsuisse Faculty, University of Zurich, Zurich, Switzerland; 3 Institute of Veterinary Pathology, Vetsuisse Faculty, University of Zurich, Zurich, Switzerland; 4 Institute of Veterinary Pathology, University of Giessen, Giessen, Germany; 5 Institute of Biomedical Engineering, University of Zurich and ETH, Zurich, Switzerland

## Abstract

**Background:**

In a cat that had ostensibly recovered from feline leukemia virus (FeLV) infection, we observed the reappearance of the virus and the development of fatal lymphoma 8.5 years after the initial experimental exposure to FeLV-A/Glasgow-1. The goals of the present study were to investigate this FeLV reoccurrence and molecularly characterize the progeny viruses.

**Results:**

The FeLV reoccurrence was detected by the presence of FeLV antigen and RNA in the blood and saliva. The cat was feline immunodeficiency virus positive and showed CD4^+ ^T-cell depletion, severe leukopenia, anemia and a multicentric monoclonal B-cell lymphoma. FeLV-A, but not -B or -C, was detectable. Sequencing of the envelope gene revealed three FeLV variants that were highly divergent from the virus that was originally inoculated (89-91% identity to FeLV-A/Glasgow-1). In the long terminal repeat 31 point mutations, some previously described in cats with lymphomas, were detected. The FeLV variant tissue provirus and viral RNA loads were significantly higher than the FeLV-A/Glasgow-1 loads. Moreover, the variant loads were significantly higher in lymphoma positive compared to lymphoma negative tissues. An increase in the variant provirus blood load was observed at the time of FeLV reoccurrence.

**Conclusions:**

Our results demonstrate that ostensibly recovered FeLV provirus-positive cats may act as a source of infection following FeLV reactivation. The virus variants that had largely replaced the inoculation strain had unusually heavily mutated envelopes. The mutations may have led to increased viral fitness and/or changed the mutagenic characteristics of the virus.

## Background

Domestic cats are natural hosts to feline leukemia virus (FeLV) [1] and feline immunodeficiency virus (FIV) [2]. These retroviruses can induce tumors and immunosuppression. While FIV-infected cats usually become persistently infected when exposed to the virus [3], the susceptibility of cats to FeLV infection varies remarkably [4]. FeLV infection has been shown to result in different outcomes, which makes FeLV-infected cats an appropriate animal model for the multifaceted pathogenesis of retroviruses [4]. Some cats develop progressive infection with persistent viremia and a lack of FeLV-specific humoral and cellular immunity [4,5], and they ultimately succumb to FeLV-associated diseases. The majority of FeLV-exposed cats develop a regressive infection with undetectable or transient viremia and an effective immune response [5]. In some of these cats, localized FeLV infections have been demonstrated [6,7]. Latent, nonproductive infection characterized by the absence of viremia and the persistence of the virus in the bone marrow can be identified in cats following regressive infection. This viral persistence can be detected by culturing bone marrow cells in the presence of corticosteroids [8-11]. The majority of cats with latent infection eliminate the virus from the bone marrow within 30 months of exposure to infection [11,12].

The development of sensitive and specific real-time TaqMan polymerase chain reaction (PCR) assays [13-15] led to the reassessment of FeLV infection outcomes [14,16-18]. In these studies, cats with progressive infection became persistently positive for the provirus and viral RNA and had high viral loads. Cats with regressive infection had lower provirus and viral RNA loads than cats with progressive infection. The provirus became undetectable over time only in a few cats with regressive infection [14].

We now report on a specific pathogen-free (SPF) cat that had been part of an early FeLV vaccination study, which was performed to test the first recombinant FeLV vaccine and to examine the influence of a preexisting FIV infection on the immune response and vaccine efficacy [19]. The cat had been infected with FIV prior to FeLV vaccination and exposure to FeLV-A/Glasgow-1. The cat developed transient FeLV viremia but was FeLV negative thereafter. After being healthy for 8.5 years, the FeLV antigen and viral RNA reappeared in the blood; FeLV was shed via the saliva; and the cat developed a multicentric FeLV-positive lymphoma. The goals of the present study were to investigate the recurrence of FeLV in this cat and to determine the molecular characteristics of the progeny viruses and their distribution in order to provide further knowledge on the molecular determinants of FeLV pathogenicity and to deepen our understanding of the host-retrovirus interaction.

## Methods

### Animal, virus exposures and long-term follow-up

**Figure 1:**
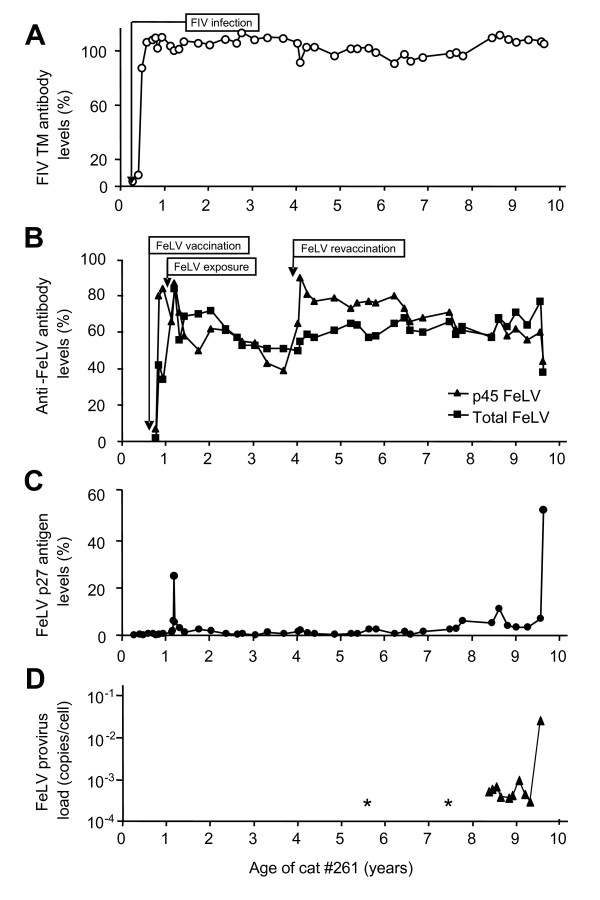
**Time course of FeLV infection in cat #261**. A) FIV transmembrane (TM) specific antibody levels as determined by ELISA. B) Total anti-FeLV antibodies (black squares) and anti-FeLV p45 antibodies (black triangles) as determined by ELISA. C) Plasma FeLV p27 antigen levels as determined by ELISA. D) FeLV provirus loads as determined by real-time PCR. Two samples, indicated by asterisks, were positive for FeLV by non-quantitative PCR. Time points of FIV infection (at the age of 17 weeks), FeLV vaccinations (41 weeks, 4 years) and FeLV exposure with FeLV-A/Glasgow-1 (59 weeks) are indicated.

A female SPF cat (cat #261; Ciba Geigy, Basel, Switzerland), which was kept under barrier conditions and housed in a group, was intraperitoneally infected with FIV at the age of 17 weeks (Fig. 1A), as described [19]. The cat was vaccinated with a recombinant FeLV p45 protein vaccine (Leucogen, Virbac, Nice, France) at the age of 41 weeks (Fig. 1B), and it was exposed intraperitoneally to FeLV-A/Glasgow-1 18 weeks later (Fig. 1B) [19]. At the age of four years (2.9 years post FeLV infection [p.i.]), the cat was revaccinated twice with the FeLV vaccine (Fig. 1B). The cat was observed for 8.5 years p.i., for a total observation period of 9.6 years. It was co-housed with FeLV p27-positive cats during the first seven years p.i., after which it was kept with p27-negative cats. The study was officially approved by the veterinary office of the Swiss Canton of Zurich (197/89, 43/90, 66/91, 131/91, 329/91, 56/95). Complete hemograms and, at selected time points, serum biochemistry analyses were performed. CD4^+ ^and CD8^+ ^cell subsets were determined by flow cytometry as described [20], starting twenty months after FIV infection at the age of two years.

### Serological assays and virus isolation

ELISA was used to detect the levels of the FeLV p27 antigen [21] and antibodies to the FIV transmembrane protein [22], total FeLV, and FeLV p45 [19,23]. ELISA results were calculated as a percentage after normalization to the positive control, which was assayed on every plate. FeLV-neutralizing antibodies were measured by a focus-inhibition assay [19]. Virus isolation was performed for FIV using blood lymphocytes [19,23] and for FeLV using heparinized plasma [19]. To detect FeLV latency, bone marrow that was collected 24 weeks p.i. at the age of 1.6 years was cultured in the presence of hydrocortisone [19].

### Necropsy

The cat underwent histopathological examination, and samples from 27 tissues (Table 1) were collected. Tissues for histology were fixed in 10% buffered formalin and processed by standard procedures. Samples for PCR analyses were snap-frozen in liquid nitrogen and stored at -70°C.

**Table 1:** Detection of lymphoma and FeLV in tissues collected upon necropsy from cat #261.

Tissue	Lymphoma (Histology)	*In situ *Hybridization		Immunohistology		
		**gp70**	**p27**	**gp70**	**p27**	**p15E**

Salivary glands:						
- Mandibular	-	-	-	-	-	-
- Parotid	-	-	-	-	+	-
Duodenum	+ in GALT	+/++	+/++	-	-	-
Jejunum	+ in GALT	-	-	-	-	-
Ileum	+ in GALT	++ in GALT	++ in GALT	-	-	-
Colon	+ in GALT	+/++	+/++	-	-	-
Rectum	+ in GALT	+	+	-	-	-
Liver	+ (mainly centro-acinous)	++	++	-	-	-
Spleen	+	nt	nt	nt	nt	nt
Thymus	+	++	++	-	-	-
Tonsil	+	+/++	+	-	-	-
Lymph nodes:						
- Sternal	+	++	++	-	-	-
- Popliteal	+	+/++	+/++	-	-	-
- Submandibular	+	+/++	+/++	-	-	-
- Mesenteric	+	+/++	+/++	-	-	-
Bone marrow	+	+	+	-	-	-
Kidney	+	+/++	++	-	-	-
Urinary bladder	+	++	++	-	-	-
Brain	-	-	-	-	-	-
Spinal cord	-	-	-	-	-	-
Ischiatic nerve	-	-	-	-	-	-
Muscle upper hind leg	-	nt	nt	nt	nt	nt
Lung	+ (foci)	+	+	-	++	-
Myocardium	-	-	-	-	-	-
Aorta	-	-	-	-	-	-
Diaphragm	+	+	+	-	-	-
Thyroid and parathyroid glands	-	-	-	-	-	-

Lymphoma diagnosed by macroscopic and histological examination and FeLV detected by *in situ *hybridization and immunohistology.  GALT = gut associated lymphoid tissue; nt = not tested; - = negative reaction; + = positive reaction; ++ = strong positive reaction; +/++ = positive or strong positive reaction depending on tissue section/cell type

### Immunohistology

FeLV proteins were detected in formalin-fixed paraffin-embedded (FFPE) tissue sections by an indirect immunoperoxidase assay (IPA) using antibodies directed against p27, gp70 and p15E as previously described [24,25]. Controls were established with a monoclonal antibody directed against an unrelated antigen. FFPE lymphoma-positive tissue sections (sternal and mesenteric lymph nodes, large intestine, spleen and liver) were tested to identify B and T cells using a CD3 T-cell marker (M7254, DAKO) and the B-cell markers for CD79 (M7051, DAKO), CD20 (RB-90-13-P, Labvision, Thermo Fisher Scientific, Fremont, USA) and CD45R [26] (clone B220 [Ly5]; Linaris, Wertheim-Bettingen, Germany) together with the ChemMate detection kit (K5003, DAKO).

### Nucleic acid extraction

DNA from 200 μL of saliva or buffy coat that was collected from EDTA-anticoagulated blood was extracted using the QIAamp Blood Mini Kit (Qiagen, Hombrechtikon, Switzerland). RNA from serum and saliva samples that were collected at the time of euthanasia was extracted using the viral RNA Mini Kit (Qiagen). Tissue samples were homogenized as described [27], and DNA was extracted using the QIAamp DNA Tissue Kit (Qiagen). RNA from tissues was purified using the ABI Prism 6700 Automated Nucleic Acid Workstation (Applied Biosystems, Rotkreuz, Switzerland) or the RNeasy Mini Kit (Qiagen). Nucleic acids were extracted from urine (200 μL) and feces (~5 mg) collected at the time of euthanasia as described [28,29]. Negative extraction controls consisting of phosphate buffered saline were included with each batch. In those experiments for which complementary DNA (cDNA) levels are given, the isolated RNA was reverse transcribed into cDNA using the High Capacity cDNA Reverse Transcription Kit (Applied Biosystems) prior to real-time PCR.

### Total FeLV provirus and viral loads

Total FeLV provirus loads were quantified by TaqMan real-time PCR (U3 region) [15]. The number of provirus copies per cell was calculated using feline glyceraldehyde-3-phosphate dehydrogenase (GAPDH) copy numbers [30,31]. Ten blood samples that were collected from 7.3 to 8.5 years p.i. (at the age of 8.4 to 9.6 years) were available for quantitative analyses. Two samples that were collected at 4.5 and 6.4 years p.i. (at the age of 5.6 and 7.5 years) were analyzed by nested FeLV PCR [13]. Tissue viral loads were determined from cDNA using the U3 real-time PCR assay and were normalized to GAPDH and ribosomal protein S7 (RPS7) cDNA copy numbers [27]. Viral RNA loads in the serum and saliva were calculated as copies per mL.

### FIV provirus and viral RNA loads

FIV provirus loads were determined by quantitative TaqMan real-time PCR [32]. FIV RNA loads were quantified using a protocol [15] and oligonucleotides previously described [32]. They were normalized according to GAPDH mRNA loads as determined by real-time PCR using a protocol [15] and previously described oligonucleotides [31]. For absolute quantification, standard RNA templates were prepared [33] from plasmids containing either FIV [34] or GAPDH [31] sequences. The standard RNA was quantified and aliquoted as described [35].

### FeLV envelope gene specific real-time PCR assays

FeLV-A/Glasgow-1 envelope gene (*env*) was quantified by TaqMan real-time PCR assay as described [5]. In addition, primers and probes for an *env *variant-specific assay were designed using Primer Express software (version 3, Applied Biosystems; Table 2). The PCR reactions were performed as described [27] using 400 nM primers, and 200 nM of fluorogenic probe (Microsynth, Balgach, Switzerland).

**Table 2:** Oligonucleotides used in this study

Assay/Application	Oligonucleotide	Sequence	Amplicon size (bp)	Nucleotide position (bp)
TaqMan^® ^PCR assay				
*env *variant^1^	Forward	GAT CCG GAC CGA CCA TAA TTA A	105	1,912 - 1,933
	Probe	TGT ATG ATT CCA TTT AGT CCC^6^		1,935 - 1,955
	Reverse	ACA CCA CTG CAG TAG CTG GCT AA		2,017 - 1,995
Production of standard				
FeLV-A/Glasgow-1^2^	Forward	TGG GGC CAA AGG GAA CAC AT	598	456 - 475
	Reverse	GTT ACC TAA GAT TGC AAT CCC TTC G		1,054 - 1,030
*env *variant^3^	Forward^5^	CCT ATG GCT CAC TTC TTT GAT ACT GAT ATC TCT A	2,664^7^	5,617 - 5,650
	Reverse	TTA TAG CAG AAA GCG CGC G		8,281 - 8,263
*In situ *hybridization^4^				
p27	Forward	TAC GCC TTT ATC GCC AGT TG	342	1,840 - 1,859
	Reverse	ATC TTT CTT CCC TTT CCT CTG G		2,181 - 2,160
gp70	Forward	AGG GAT TGC AAT CTT AGG TA	219	6,952 - 6,971
	Reverse	TTA CAG GCC CAA TAG GTG		7,170 - 7,153

^1 ^based on FeLV SP261-III [GenBank: EU359305]; ^2 ^based on FeLV-A/Glasgow-1 [GenBank: M12500]; ^3 ^based on FeLV-A Rickard strain [GenBank: AF052723], ^4 ^based on FeLV-FAIDS [GenBank: M18247]; ^5 ^[29]; ^6 ^5' FAM/3' TAMRA; ^7 ^the PCR product included the first 300 nucleotides of the LTR in addition to *env*

Linearized plasmid DNA containing the appropriate envelope gene sequence (Table 2) was used as a standard template to test the specificity and sensitivity of the two *env*-specific real-time TaqMan assays and for absolute quantification. Copy numbers were determined spectrophotometrically (Nanodrop^® ^ND-1000, Witec, Littau, Switzerland), and ten-fold serial dilutions were prepared as described [33]. The sensitivity of the system was determined by an endpoint dilution experiment [33]. The specificity was tested further with an endogenous FeLV sequence standard containing 10^8 ^copies/reaction [36] and with DNA from three SPF cats.

### Detection of FeLV subgroups

FeLV-subgroups were investigated in the kidney, spleen, rectum, diaphragm, thymus, mandibular gland and myocardium by conventional PCR using the FeLV-A specific primers RB59 and RB17, the FeLV-B specific primers RB53 and RB17 and the FeLV-C specific primers RB58 and RB47 as described [37,38].

### *In situ *hybridization

Digoxigenin-labeled RNA probes recognizing gp70 and p27 were used for *in situ *hybridization [39]. The probes were constructed from FeLV-A [GenBank: M18247] [40] using the primers listed in Table 2. The PCR products were cloned using the TOPO TA Cloning kit (Invitrogen BV, Groningen, The Netherlands). *In vitro *reverse transcription of the linearized plasmids and digoxigenin labeling was performed using the DIG RNA Labeling Kit (Roche Diagnostics GmbH, Mannheim, Germany). Positive strand RNA was used as a negative control. Hybridized digoxigenin was visualized with 150 U of anti-digoxigenin AP Fab fragments (Roche Diagnostics GmbH) and nitroblue tetrazolium chloride/5-bromo-4-chloro-3-indolyl phosphate.

### Sequencing of the *env *and long terminal repeat (LTR) regions of FeLV progeny viruses

For the analysis of the full-length FeLV *env *sequences, DNA from the kidney and spleen was amplified as described [29] using *env *variant primers yielding a 2'664 bp product (Table 2). PCR products were either sequenced directly (Synergene Biotech GmbH, Schlieren, Switzerland) or after TOPO TA cloning. Three *env *variants were identified: KI261-I from the kidney (direct sequencing), KI261-II from the kidney (direct sequencing) and spleen (2 clones) and SP261-III from the spleen (19 clones).

Full-length U3 regions of the 3' LTR were amplified using the forward primer CAA TAC GAT CCG GAC CGA CCA TG and the reverse primer CGG GGC GGT CAA GTC TCG GCA AAG (adapted from [41]). PCR products (446 bp) were cloned as above. A total of 18 FeLV LTR clones, including 7 from the kidney, 3 from the bone marrow, 3 from the liver and 5 from the spleen, were sequenced (Microsynth).

### Phylogenetic analyses

Phylogenetic and molecular evolutionary analyses were conducted using MEGA version 4 [42]. The FeLV surface unit (SU) and the LTR sequences were aligned using CLUSTAL W [43]. For SU sequences, bootstrap support (1,000 replicates) was calculated by the neighbor-joining (NJ), minimum evolution (ME) and maximum parsimony (MP) methods, and results > 70% were considered to be significant [44]. MP trees were obtained using the Close-Neighbor-Interchange algorithm [45] with search level three [44], in which initial trees were obtained by the random addition of sequences (10 replicates). All positions containing gaps and missing data were eliminated from the dataset (complete deletion option).

### Assessment of clonality in the lymphoma

To assess the clonality in the lymphoma from cat #261, the variable region genes of the feline immunoglobulin heavy chain (IGHV) and the T-cell receptor gamma (TCRG) were analyzed using PCR for antigen receptor gene rearrangements (PARR). To this end, DNA extracted from snap-frozen tissues was evaluated as described [46,47]. In addition, FFPE samples were analyzed without preceding DNA extraction: single 10 μm sections were treated with 20 mg/mL of proteinase K in 150 μL 1× Phusion HF Reaction Buffer (BioCat, Heidelberg, Germany) at 60°C overnight. After inactivation of the proteinase and centrifugation at 16,000 × g for 2 minutes, the fluid phase was used to confirm the DNA quality as described [48] with Phusion DNA Polymerase (BioCat). The samples were assayed by IGHV PARR analysis as described [48] with the following modifications: sense primers with annealing sites within the same framework region (1 and 3) were combined in one reaction (V1FR1 and V3FR1; V1FR3 and V3FR3).

### Statistics

Statistical analyses were performed with GraphPad Prism for Windows (version 4.03, GraphPad software, San Diego, CA). Differences among three or more groups were analyzed by Kruskal-Wallis one-way ANOVA by Ranks (p_KW_) and Dunn's post test. Differences between two groups were tested for significance using the Mann-Whitney U-test (p_MWU_). Differences were considered significant if p < 0.05. Observed agreement, expected agreement and Cohen's kappa coefficient were calculated as described [49], with kappa values interpreted as suggested [50].

### Nucleotide sequence accession numbers

The sequences described were submitted to GenBank: *env *[EU359303 to EU359305] and LTR [FJ613291 to FJ613296].

## Results

### Long-term follow-up

**Figure 2:**
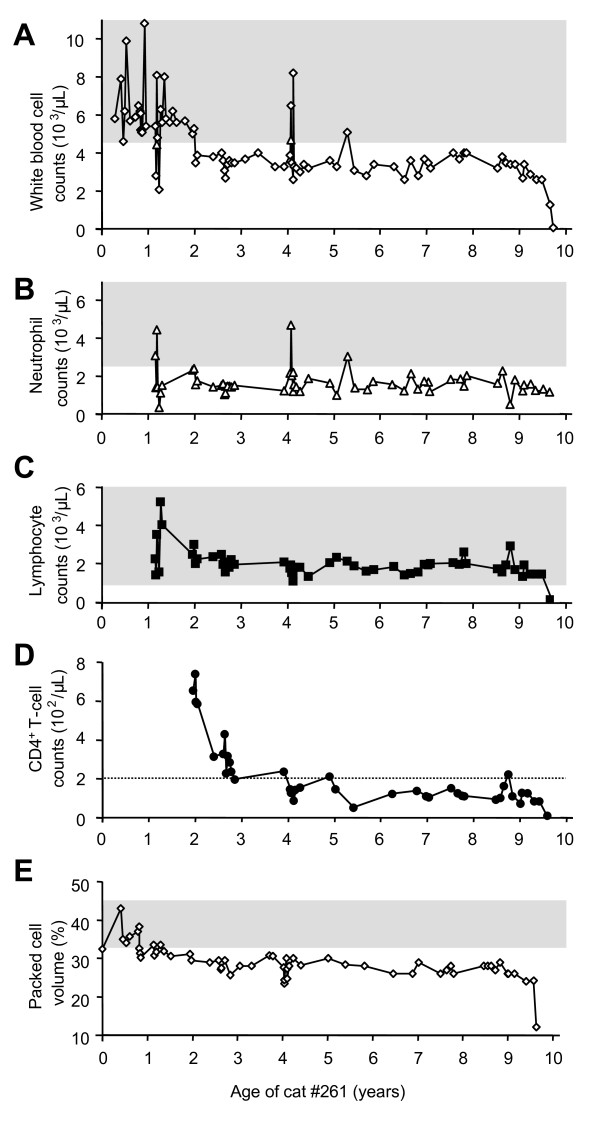
**Time course of hematological parameters for cat #261**. A) White blood cell counts. B) Neutrophil granulocyte counts. C) Lymphocyte counts. D) CD4^+ ^T-cell counts. E) Packed cell volume. The reference ranges (5th to 95th percentiles) are indicated by the shaded areas (A to C, and E). No reference range was available for the absolute numbers of CD4^+ ^T cells. In panel D, the dotted line indicates a CD4^+ ^T-cell count of 200 CD4^+ ^T cells/μL. No differential was possible at the time of sacrifice due to the low WBC number (100 cells/μL).

We analyzed blood samples collected from cat #261 over an observation period of 9.6 years as well as tissue samples collected at necropsy to investigate the FeLV recurrence and the development of virus variants in a long-term FIV-infected, FeLV provirus-positive, antigen-negative cat. The cat remained FIV-infected throughout the entire study (Fig. 1A), and all blood samples tested were FIV provirus-positive. Subsequent to the FeLV exposure at the age of 1.1 year, the cat developed transient FeLV viremia. FeLV virus was isolated from the blood in week 2 p.i., and the cat was p27-positive in week 3 p.i. (Fig. 1C). Transiently decreased white blood cell (WBC) counts were observed in weeks 1 and 4 p.i. (Fig. 2A), and the cat developed neutropenia (Fig. 2B). High neutralizing antibody titers were demonstrated by 12 weeks p.i., and total anti-FeLV antibody levels were persistently high (Fig. 1B). The virus could not be isolated from the bone marrow. Revaccination against FeLV at the age of four years yielded a marked boost in anti-p45 antibodies (Fig. 1B) and temporary increases in the WBC count and the number of neutrophils (Fig. 2A and 2B). There was no increase in the level of FeLV p27 (Fig. 1C). All tested blood samples were FeLV provirus-positive (Fig. 1D).

### Development of disease

At 8.5 years p.i., at the age of 9.6 years, cat #261 became anorexic and lost weight, which was followed by dyspnea, dehydration, pale mucous membranes and a painful abdomen upon palpation. Severe non-regenerative anemia (packed cell volume: 12%; Fig. 2E; hemoglobin 3.8 g/dL) and severe leukopenia (100 cells/μL; Fig. 2A) were observed. Due to the low WBC count no differential was possible at this time. The last WBC differential performed three weeks prior to sacrifice revealed neutropenia (1,066 cells/μL Fig. 2B) and severe lymphopenia (182 cells/μL; Fig. 2C). In addition, CD4^+ ^T cells were depleted (9 cells/μL; Fig. 2D). At the time of euthanasia, the animal had elevated levels of blood urea (37.6 mmol/L), creatinine (227 μmol/L), calcium (3.8 mmol/L), potassium (6.3 mmol/L) and phosphorus (2.9 mmol/L). The urine had a specific gravity of 1.016, and the protein-creatinine quotient (3.50) was elevated. Upon necropsy, lymphoma was detected in 18 out of 27 tissues by histological examination (Table 1, Additional file 1).

### Reoccurrence of FeLV

Simultaneously with disease development, the cat became FeLV p27 antigen-positive (Fig. 1C; at the age of 9.6 years). A marked decrease in FeLV-specific antibodies was noted (Fig. 1B). FeLV RT-PCR analysis of the serum was positive (mean load: 2.8 × 10^4 ^copies/mL serum), and a saliva sample was positive for FeLV p27 (11%; values above 4% are considered positive [28]) and FeLV viral RNA (1.2 × 10^7 ^copies/mL saliva).

### Characterization of lymphoma

A diffuse proliferation of small to predominantly medium sized lymphatic cells was observed. The nuclei were mostly round, and had a finely to coarsely stippled chromatin. One single central nucleolus or multiple randomly distributed nucleoli were seen (Additional file 1C). Between 10% (liver) and 100% (sternal lymph node) of the assessed tissues were affected. Based on the histological appearance and according to the WHO Histological Classification of Hematopoietic Tumors of Domestic Animals [51] a diffuse large B-cell lymphoma was diagnosed. The B-cell lineage was confirmed by positive staining in immunohistochemistry using the anti-CD20 and anti-CD45R B-cell antibodies (Additional file 1D). Anti-CD3 T-cell and anti-CD79 B-cell staining were negative. PARR analysis of the IGHV using FFPE tissues (liver, intestine, lymph node combined) with the framework region 3-specific primers showed a reproducible intensive band in the expected size range (~180 bp) upon heteroduplex analysis; a second weak band was non-reproducible. These results were corroborated by the investigation of snap-frozen tissues from spleen for rearrangements in the IGHV: a reproducible clonal PCR product of the expected size (~140 bp) within a background polyclonal smear was detected. No rearrangement of the TCRG gene was observed. Accordingly, the lymphoma was categorized as a monoclonal proliferation of B cells.

### Presence of FeLV and FIV in the lymphoma

Using IPA, FeLV p27-specific reactions were detected in 2 out of 25 tested samples, while FeLV p27 and gp70-specific gene sequences were found in 16 out of 25 samples by the more sensitive *in situ *hybridization (Table 1). When the results of the histological detection of lymphoma and FeLV *in situ *hybridization were compared, we observed an agreement of 96% (expected agreement 55%) and a Cohen's kappa value of 0.91, indicating almost perfect agreement between these two analyses.

**Figure 3:**
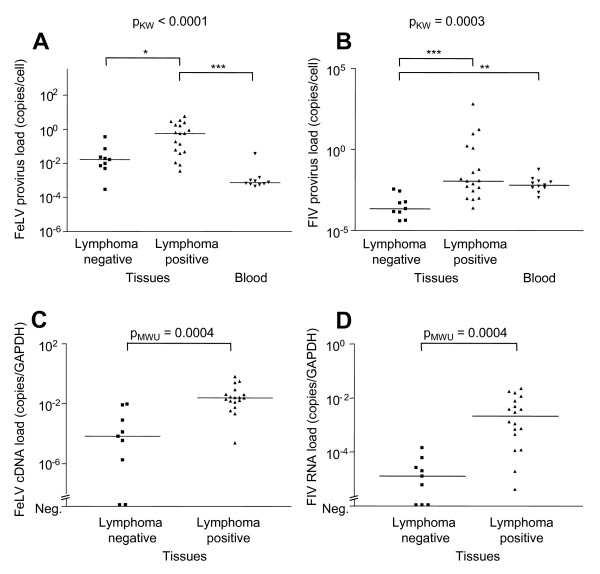
**FeLV and FIV provirus and viral loads in blood and tissue samples from cat #261**. FeLV and FIV provirus and viral loads in cat #261, quantified in the various tissues collected upon necropsy and in blood samples collected over the course of the last 14 months prior to sacrifice. Provirus and cDNA loads were determined using TaqMan real-time PCR and viral RNA loads were measured by TaqMan real-time RT-PCR. The tissues were classified according to the absence (n = 9) or presence (n = 18) of apparent lymphoma, as determined by histological examination. A) FeLV provirus loads. B) FIV provirus loads. C) FeLV viral (cDNA) loads in the tissues. D) FIV viral loads in the tissues. Viral tissue loads were normalized using GAPDH. Provirus loads (A and B) were tested for statistical differences by Kruskal-Wallis one-way ANOVA by Ranks (p_KW _as indicated) and subsequently by Dunn's post test: * = p < 0.05; ** = p < 0.01; *** = p < 0.001. Viral loads (C and D) were tested for statistically significant differences using the Mann-Whitney U-test (p_MWU _as indicated).

All 27 tissues examined were FeLV and FIV provirus-positive. FeLV provirus loads were significantly higher in tissues than in the blood (p_MWU _< 0.0001), and they were significantly higher in tissues with lymphoma than in those without lymphoma (Fig. 3A). FIV provirus loads in tissues with lymphoma were higher when compared to the loads in healthy tissues, but they were not higher than the loads in the blood (Fig. 3B).

Out of the 27 tissues tested, 93% were positive for FeLV transcription, and 89% were positive for FIV transcription. Tissues with lymphoma had significantly higher FIV and FeLV viral loads than healthy tissues (Fig. 3C and 3D).

### Characterization of FeLV progeny viruses

No FeLV-B (recombination with endogenous FeLV sequences) and FeLV-C subtypes were detected by conventional PCR. Three heavily mutated FeLV-A *env *variants were identified that showed 89-92% amino acid identity with each other and the highest, albeit modest, resemblance to FeLV-A/Glasgow-1 (for details see Table 3). Sequence variations were scattered throughout *env *and included point mutations in functional domains, such as the variable regions (VRA, VRB and VRC) and the proline rich region (PRR; [52]; for details see Additional file 2). All potential disulfide bonds were conserved in the three *env *variants compared to FeLV-A/Glasgow-1, as was the PHQ motif that is located in the N terminus of the receptor-binding domain and is critical for triggering virus fusion [52,53]. In addition, 11 out of 13 potential N-linked glycosylation sites were conserved when compared to FeLV-A/Glasgow-1, with two sites being lost. In addition, three new potential N-linked glycosylation sites were identified.

**Table 3:** Sequence comparison of the *env *variants with prototype FeLV-A, -B, and -C

Amino acid identity (%)	FeLV-A/Glasgow-1 **[GenBank: **M12500]	FeLV-B/Gardner-Arnstein **[GenBank: **K01209]	FeLV-C/Sarma **[GenBank: **M14331]
*env*	89-91	76-77	85-86
SU	86-89	68-69	81-83
VRA	68-74	29-34	47-53
VRB	48-69	N.A.	40-63

N.A. = Not applicable (< 20%)

**Figure 4:**
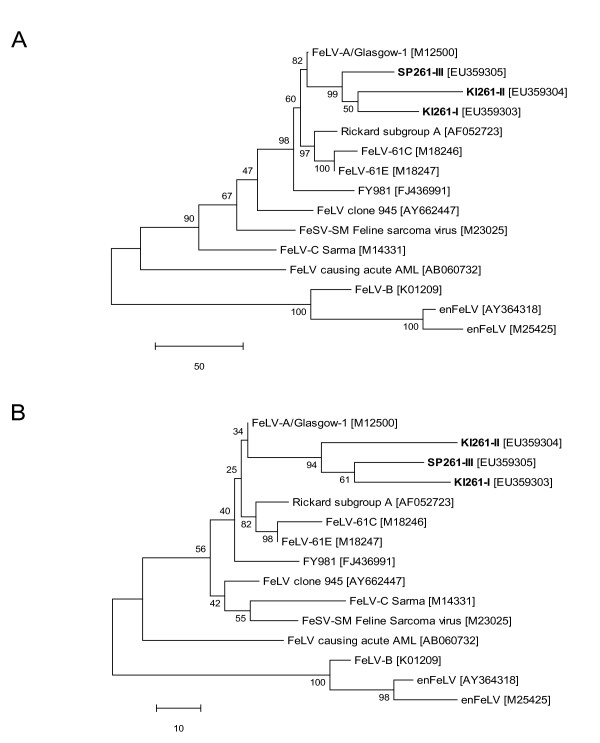
**Evolutionary relationship of the three SU variants found in cat #261**. Phylogenetic trees were constructed by the MP method. Trees were drawn to scale, with the length being relative to the number of changes over the entire sequence. The percentage of replicate trees in which the associated taxa clustered together in the bootstrap test (1,000 replicates) is shown next to the branches. A) Relationships at the DNA level. MP tree length: 765. The codon positions included were 1st + 2nd + 3rd + noncoding. There were a total of 1,363 base positions in the final dataset of which 349 were parsimony-informative. B) Relationships at the protein level. MP tree length: 329. There were a total of 453 amino acid positions in the final dataset of which 121 were parsimony-informative. GenBank accession numbers of the sequences included in the phylogenetic analyses are noted in square brackets following the virus identity. SP261-III, KI261-I and KI261-II (depicted in bold) were derived from cat #261.

Phylogenetic analysis conducted by the MP, NJ and ME methods revealed the presence of a cluster of the three *env *progeny variants. In the trees based on nucleotide as well as protein sequences, the *env *variant sequences were most closely related to the original challenge strain FeLV-A/Glasgow-1 (Fig. 4).

Sequencing of U3 led to nine groups of LTR sequences (Additional file 3). An overall U3 sequence conservation of 95-97% was found when the full-length progeny LTR sequences were compared to FeLV-A/Glasgow-1. Point mutations were found at 31 locations in the LTR sequence between the start of U3 and the TATA box. Several changes were found within the enhancer framework that comprises the binding sites for the transcription factors, the leukemia virus factor b (LVb), simian virus 40 core enhancer (CORE), nuclear factor 1 (NF1), glucocorticoid response element (GRE) and the FeLV-specific binding motif (FLV-1). There was one insertion and one transition found in the LVb binding site (Additional file 3). Four clones had a point mutation in the CORE. Mutations were found at two locations within the NF1 binding site, and one of these was detected in all of the clones. One mutation was located in the GRE binding motif, and two clones had a mutation in the FLV-1. Additional mutations were detected at 24 locations outside of these domains, with the majority of these mutations located upstream of the LVb site. No duplications of the enhancer or the upstream region of the enhancer (URE) were detected in any of the clones that were investigated.

### Dominance of *env *variants

Using standard DNA templates, real-time PCR assays for the variant and FeLV-A/Glasgow-1 *env *were shown to be specific for the respective sequences, without amplifying endogenous FeLV sequences. The detection limit of both assays was one copy/PCR, and the amplification efficiency was 99% for FeLV-A/Glasgow-1 and 98% for the variant.

**Figure 5:**
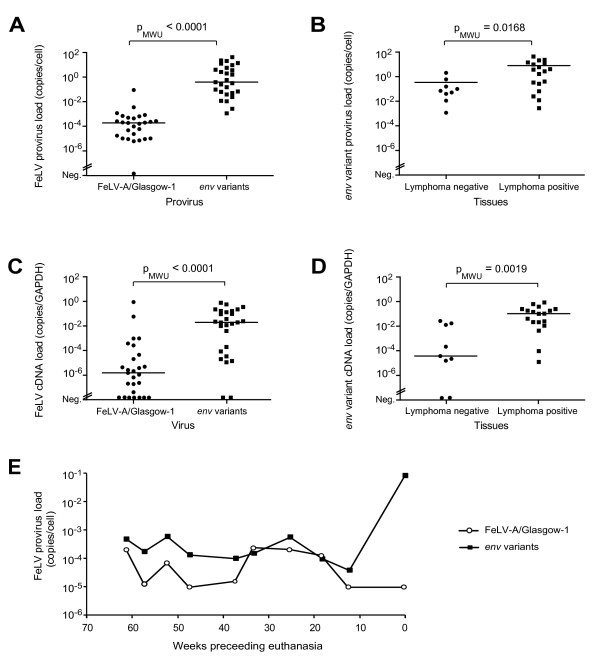
**FeLV-A/Glasgow-1 and *env *variant loads in blood and tissue samples from cat #261**. Loads of FeLV-A/Glasgow-1 and the *env *variants of cat #261 quantified by real-time PCR in the tissues collected upon necropsy (A to D) and in blood samples collected over the course of the last 14 months prior to sacrifice (E). A) Provirus loads of FeLV-A/Glasgow-1 and *env *variants in all tissues. B) *env *variant provirus loads in tissues without (n = 9) and with (n = 18) apparent lymphoma. C) Viral (cDNA) FeLV-A/Glasgow-1 and *env *variant loads in all tissues. D) Viral (cDNA) *env *variant loads in tissues with and without apparent lymphoma. E) Time course of provirus loads of FeLV-A/Glasgow-1 and *env *variants in the blood of cat #261. Viral loads (C and D) were normalized using GAPDH. Provirus and viral loads (A to D) were tested for statistical significance using the Mann-Whitney U-test (p_MWU _as indicated).

Provirus of the *env *variants was identified in all 27 tissues, and the FeLV-A/Glasgow-1 *env *provirus was found in 26 out of 27 tissues (Fig. 5A; for details see Additional file 4A). With the exception of the duodenum, the provirus loads of the *env *variants were higher in every tissue than the FeLV-A/Glasgow-1 *env *provirus loads, and, when the results from all tissues were combined, a significant difference was found (p_WMU _< 0.0001, Fig. 5A). Remarkably, the provirus loads of the *env *variants were also significantly higher in tissues with lymphoma than in tissues without lymphoma (p_MWU _= 0.0168, Fig. 5B), while no significant difference was found in provirus loads between these two groups for FeLV-A/Glasgow-1 (data not shown).

Concordant with these observations, the viral RNA loads of the *env *variants were higher than the FeLV-A/Glasgow-1 viral RNA loads in all but three tissues, and overall the variant *env *RNA levels were significantly higher than those of FeLV-A/Glasgow-1 (p_MWU _< 0.0001, Fig. 5C and for details see Additional file 4B). Moreover, viral RNA levels of the *env *variants were significantly higher in tissues with lymphoma than in healthy tissues (p_MWU _= 0.0019, Fig. 5D), whereas no difference was detectable for the levels of FeLV-A/Glasgow-1 *env *RNA (data not shown and Additional file 5D). These results were confirmed using the ribosomal protein S7 (RPS7) as an additional reference gene for the normalization of expression (Additional file 5). RPS7 was among the most stable reference genes tested previously [27].

When blood samples collected over the course of the last 14 months prior to sacrifice were analyzed, all samples tested provirus positive for the *env *variant and FeLV-A/Glasgow-1. An increase in the *env *variant provirus load (> 100×) was observed at the time of FeLV reoccurrence, while the FeLV-A/Glasgow-1 provirus load remained low (Fig. 5E). No RNA samples from blood were available for detailed RT-PCR analyses. The FeLV-positive saliva sample collected at the time of sacrifice, tested viral RNA positive for FeLV-A/Glasgow-1 but negative for the *env *variants. The feces and urine collected at the same time point were positive for *env *variants.

## Discussion

We demonstrate the reappearance of FeLV in an ostensibly recovered cat 8.5 years after the initial virus exposure. The SPF cat under investigation was kept in a controlled environment and was monitored closely throughout the entire observation period. It was exposed to a specified prototype FeLV-A strain, FeLV-A/Glasgow-1, after which a regressive infection with clearance of viremia was demonstrated. Latent FeLV infection, which is defined as replicating virus in bone marrow cell cultures supplemented with corticosteroids [8-10], was undetectable 24 weeks p.i. (at the age of 1.6 years); additional samples for testing were unavailable. All blood samples collected during the last 14 months before sacrifice were FeLV provirus-positive and the provirus loads in the peripheral blood were low, a characteristic of FeLV provirus-positive antigen-negative cats [13]. Only at the age of 9.6 years and concurrent with the reoccurrence of FeLV antigenemia, the blood FeLV provirus loads increased.

The cat was co-infected with FIV. This virus infection is frequently used as an animal model for retroviral diseases and in particular AIDS due to the common genus and the similarities with the human and simian immunodeficiency virus (HIV, SIV). The Swiss FIV isolate used in this study, FIV Z Ga, led to an AIDS-like syndrome characterized by complete CD4^+ ^depletion, similar to what has been described for FIV Petaluma [2] and HIV. The breakdown of the immune system in cat #261, as a result of end-stage FIV infection, and the resulting loss of the surveillance of FeLV replication was probably crucial for the reoccurrence of FeLV. Reactivation of FeLV infection upon experimental immune suppression in aviremic cats has been reported [9-11,17]. Some of these cats received high doses of corticosteroids for several weeks to provoke FeLV reactivation. The potential for reactivation seemed to be associated with the FeLV isolate [11], and the probability of reactivation decreased with increasing duration after FeLV exposure. Most cats were free of reactivatable virus several months after clearance of FeLV viremia or after the removal from constant challenge [11,12]. In cat #261, the last potential exposure to FeLV was 18 months prior to FeLV reactivation. So far, in the absence of iatrogenic/experimental immune suppression, only a limited number of cases of reactivation of FeLV infection have been reported [7,11,14,17]. In some of these cats, natural stress was thought to be the initiator of reactivation [11]. To the best of our knowledge, this is the first documentation of FeLV reoccurrence in a cat with immunosuppression due to FIV infection.

Concurrent with the reappearance of FeLV, the FIV-infected cat had developed a fatal multicentric lymphoma. In general, lymphoma is a well-known manifestation of FeLV infection and is commonly associated with variant viruses [1,54-57]. In cat #261, the emergence of several distinct FeLV variants has been demonstrated. Moreover, lymphoma has also been described in FIV and FeLV co-infected cats [54,58]. As a result of the co-infection in cat #261, the role of FeLV and FIV in the tumorigenesis could not be easily assessed. Using specific real-time PCR assays, the FeLV progeny *env *variant loads were quantified relative to the load of the originally inoculated FeLV-A/Glasgow-1. At the time of euthanasia, the progeny viruses were more abundant than FeLV-A/Glasgow-1, thereby largely replacing the originally inoculated virus. Moreover, the *env *variant viruses were present in higher numbers in lymphoma-positive tissues than in healthy tissues. This was not the case for FeLV-A/Glasgow-1. These results may indicate that the FeLV progeny viruses found in cat #261 were relevant to the course of disease progression. Nonetheless, a strong association was also demonstrated between the presence of lymphoma and the provirus and viral RNA loads of FIV in the affected tissues.

The lymphoma was subsequently further characterized using immunohistology and PARR analysis and was, based on these results, categorized as a monoclonal proliferation of B cells. B-cell lymphomas have been reported to be the predominant form occurring during infections with FIV [59-62]. This is in parallel to HIV and SIV, where the majority of the lymphomas occurring in infected individuals were of B-cell origin [63,64]. In FeLV infection both T and B-cell lymphomas, with a predominance of T-cell lymphomas, have been described [65,66]. In the present case, FIV may have played an indirect role in tumorigenesis via immune suppression, which may have led not only to reactivation of FeLV infection but, independently, favored the emergence of a tumor cell clone. The role of the immune dysfunction induced by FIV for lymphomagenesis has been recognized earlier [67]. FIV-positive cats with lymphoma were reported to have substantially decreased CD4^+ ^T cells, a reduced cell-mediated immunity and B-cell hyperactivity [60,67]. This parallels observations for other lentiviruses. HIV-1 is generally considered to play an indirect role in tumor development via immune suppression and co-factors, such as polyclonal B-cell activation; and the involvement of other infectious agents, notably the Epstein-Barr virus, have been identified [68]. Only rarely FIV seemed to have a direct mutagenic potential [62]. Accordingly, FIV as well as HIV and SIV proviruses have been reported to be only seldom integrated in the host genome [62,67]. This is in contrast to directly oncogenic retroviruses including FeLV. In the present study, tumor tissues were positive for FIV, but since the cat was viremic, the PCR signal could have resulted at least partially from FIV positive circulating blood cells.

The outcome of an FeLV infection may depend on the age and genetic disposition of the cat, as well as on the virus genotype [4]. FeLV-A is thought to be minimally pathogenic but highly infectious and present in all natural FeLV infections [69,70]. FeLV-B, -C and -T develop within the FeLV-A infected cat via mutation, insertion or recombination events and are associated with the development of disease [71]. Using subgroup-specific PCR, only evidence of FeLV-A, but not of any other FeLV subgroups was found in cat #261. The inability to detect FeLV-B indicates that no recombination with endogenous FeLV sequences had taken place. So far reported FeLV-A isolates obtained from domestic and wild cats over several decades and across the world were found highly conserved, sharing over 97% amino acid sequence identity in the SU gene [29,40]. Remarkably, the SU sequences from the FeLV in cat #261 showed only 86-89% amino acid identity to FeLV-A/Glasgow-1. This divergence clearly exceeds that so far found among the members of the FeLV-A subgroup. Phylogenetic analysis confirmed that the closest relationship was to the original challenge strain, FeLV-A/Glasgow-1. At the time of sacrifice, virus isolation for the detection of replicating virus was not performed; therefore, receptor interference was not tested. The VRA sequence predominantly determines the receptor specificity [72]. The VRA of the progeny viruses revealed the highest resemblance to the VRA of FeLV-A/Glasgow-1; nonetheless a remarkable number of changes were observed. Recently, small changes in the VRA were shown to yield an envelope protein that was capable of using a different receptor [73,74]. However, these studies were conducted using constrained peptides in the VRA that were different from the naturally occurring FeLV isolates, and the engineered viruses were capable of using receptors outside of those traditionally used by the FeLV-A, -B and -C viral interference groups [73-75].

In gamma retroviruses, the U3 region of the LTR is a particularly potent viral determinant for pathogenicity. Changes in the FeLV U3 sequence, including the URE and the enhancer itself, drastically influence viral pathogenicity [41,76]. In the case of FeLV-related non-T-cell malignancies or non-neoplastic diseases, only one copy of the enhancer is typically found, but multiple copies of other regions of the LTR, such as the URE, have been reported [41,77]. FeLV that has been isolated from thymic lymphomas, however, contained duplications of either a partial or the entire enhancer sequence [78-80]. In FeLV-945, a natural isolate from a cat with a multicentric lymphoma, a 21-bp tandem triplication downstream of a single copy of the enhancer was shown to confer a replication advantage and to accelerate its disease onset [81]. The U3 sequences detected in cat #261 did not contain any duplication of the enhancer sequence or any repeats in the URE. However, point mutations at 31 locations were identified, including mutations in the enhancer region. Interestingly, 12 mutation sites had been described previously in FeLV-infected cats with lymphoma [41,77-79], and three additional mutations had been found in other kinds of tumors [41,77]. These mutations included one point mutation each in the CORE, the NF1 and the FLV-1 sequences. Thus, the mutations found in the U3 region of the progeny viruses in cat #261 may be causatively linked to the induction of the neoplastic disease.

The particularly high divergence of the progeny viruses from the originally inoculated FeLV-A/Glasgow-1 found in cat #261 may be explained by the long period (8.5 years) during which the virus had time to evolve in this cat. This, in turn, may indicate that minimal viral replication, at a level below the detection limit, had occurred in cat #261. In cats that have ostensibly recovered from FeLV viremia, we have found an association between plasma viral RNA, as a probable indicator of minimal viral replication in a sequestered tissue, and FeLV reactivation and tumor development [17]. No samples were available from cat #261 to determine plasma viral RNA throughout the infection. However, antigens expressed at very low levels would have constantly boosted the specific immunity, which may be in agreement with the persistently high anti-FeLV antibodies that were detected in cat #261. The long observation period in cat #261 exceeds the lifespan of most FeLV-infected pet cats, which often succumb within a few years, e.g. due to secondary infections. Since cat #261 was kept under barrier conditions, the risk of opportunistic infections or accidents was reduced.

At the time of FeLV reoccurrence, cat #261 was shedding FeLV via the saliva and had, therefore, become a potential source of infection for other cats. Shedding of FeLV RNA in the saliva has been demonstrated to be a consistent feature in antigenemic cats [28], and is thought to be the main transmission route for FeLV [82]. Interestingly, the saliva sample tested negative for the presence of *env *variants. In contrast, urine and feces were positive, albeit at a low level, for the *env *variants, indicating that the progeny viruses were shed via these secondary routes. It needs to be noted that the gastrointestinal and urinary tract, but not the salivary glands, were lymphoma positive and particularly the urinary tract showed high *env *variant provirus and viral loads whereas the mandibular gland had high FeLV-A/Glasgow-1 viral loads (Additional file 4). We subsequently tested the cats from the same cohort and co-housed with cat #261, but none of them tested provirus positive for the *env *variants. The fact that no transmission of the *env *variants was detectable could be due to the low loads of the *env *variants in urine and feces and the presumably only very short duration of shedding. Alternatively, the variants may only inefficiently or not be transmittable. Viruses selected for their replication fitness within a host during long-term infections may differ from those that are efficaciously transmitted between hosts.

## Conclusions

This is the first study to document the reoccurrence of FeLV in a cat with immunosuppression due to FIV infection many years after the initial virus inoculation. Since FIV is highly prevalent in some countries, we postulate that this phenomenon could also be observed in the field. Our results indicate that both FeLV and FIV were important for the disease development in the cat under investigation. While FIV may have mainly contributed via an immunosuppressive effect, FeLV and/or FIV may have been causally linked with the tumorigenesis. Cat #261 developed an active FeLV infection and was shedding FeLV at the time of euthanasia; thus at least part of the FeLV provirus had remained full-length and replication competent throughout the long aviremic phase. Moreover, we quantified for the first time viral loads of the FeLV challenge strain and the evolved progeny variants using sensitive, discriminating real-time PCR assays. The virus variants had largely replaced the inoculated prototype FeLV-A over time. Molecular characterization of the progeny viruses revealed a high variance in *env *not commonly found in the otherwise highly conserved FeLV-A subgroup. The large number of mutations may have led to increased viral fitness and/or changed the mutagenic characteristics of the virus.

## Competing interests

The authors declare that they have no competing interests.

## Authors' contributions

AKHH performed and analyzed the research and drafted the manuscript. VC participated in the assay design and data analysis and revised the manuscript. FSB was the veterinarian in charge for this cat and conducted *ex vivo *sample collections. PO performed the necropsy, the macroscopic and histological analysis, the sample collection and revised the manuscript. PG performed histological and part of the immunohistochemical analyses. MR performed the IPA and *in situ *hybridization. MH performed part of the immunohistochemical and PARR analyses. EB performed molecular assays. KBP was responsible for the housing and the animal care. EN and EH performed the flow cytometry experiments. HL contributed to the study design and supervised the initial study. RHL conceived and supervised the follow-up study and edited the manuscript.

## Supplementary Material

Additional file 1 **Multicentric lymphoma**. Lymphoma detected upon necropsy. A) Kidney. B) Spleen. C) Histology of sternal lymph node: diffuse proliferation of mainly medium sized lymphatic cells with round nuclei, coarsely stippled chromatin and one to multiple medium sized nucleoli. Hematoxylin and Eosin, bar = 10 μm. D) Liver: positive immunohistochemical labeling of periportal infiltrating neoplastic cells for CD45R. Avidin-biotin complex method; Papanicolaou's hematoxylin counterstain, bar = 50 μm. Click here for file

Additional file 2 **Amino acid alignment of FeLV *env *sequences**. Amino acid alignment of the *env *coding region from three FeLV subtypes (FeLV-A/Glasgow-I [GenBank: M12500], FeLV-B/Gardner-Arnstein [GenBank: K01209] and FeLV-C/Sarma [GenBank: M14331]) and the three *env *variants (KI261-I, KI261-II and SP261-III). The start of the SU region, the transmembrane domain (TM), the variable regions VRA, VRB and VRC, the PRR and the C2 disulfide-bonded loop (S-S) are labeled (according to [52]). Circles and stars represent amino acid sequences containing Asn-X-Ser/Thr, which indicate possible sites of N-glycosylation, as previously described [53,83]. Potential N-linked glycosylation sites that are conserved in FeLV-A/Glasgow-1 and all *env *variants are represented by filled circles. New potential N-linked glycosylation sites in the *env *variants that were not present in the challenge strain FeLV-A/Glasgow-1 are labeled with filled stars; those that were present in FeLV-A/Glasgow-1 but lost in the *env *variants are marked with empty stars. Dots represent identical residues, and dashes represent spaces, which were introduced for proper alignment. Click here for file

Additional file 3 **Comparison of nucleotide sequences of the FeLV U3 region**. Nucleotide sequence comparison of the U3 region from FeLV-A/Glasgow-1 [GenBank: M12500] and the progeny virus variants retrieved from cat #261. Sequences found in multiple clones were depicted once. KI261-I was found in clones from the kidney. SP261-III, pKH11.8 and pKH11.9 were found in clones from the spleen. All other listed sequences were found in at least two different tissues, including the kidney, liver, spleen, and bone marrow. Enhancer elements and their corresponding nucleotide sequences are marked in the reference strain FeLV-A/Glasgow-1 [GenBank: M12500]. Nucleotides differing from the originally inoculated strain are indicated. Primer sequences for PCR [41] were located at positions -415 to -392 and 9 to 32 (not included in the figure). Previously described mutations are indicated by numbers in brackets: (1) Jackson et al., 1996; (2) Nishigaki et al., 1997; (3) Fulton et al., 1990 and (4) Matsumoto et al., 1992. Numbers at the top of the sequence indicate nucleotide positions relative to the presumptive RNA cap site in the FeLV/Glasgow-1 LTR [53]. Grey dashed lines indicate unknown sequences. Click here for file

Additional file 4 **FeLV-A/Glasgow-1 and *env *variant provirus and viral loads in the tissues from cat #261**. A) Provirus loads of FeLV-A/Glasgow-1 and the *env *variants. B) Viral (cDNA) loads of FeLV-A/Glasgow-1 and *env *variants. Viral tissue loads were normalized to GAPDH (top) and to RPS7 cDNA copy numbers (bottom). Tissues with apparent lymphoma are indicated by shaded areas. Click here for file

Additional file 5 **FeLV viral loads in tissues from cat #261 normalized to RPS7**. A) Total FeLV viral (cDNA) loads (U3 region PCR) in tissues with and without apparent lymphoma (analogous to Fig. 3C). B) Viral (cDNA) loads of FeLV-A/Glasgow-1 and *env *variants (analogous to Fig. 5C). C) Viral (cDNA) loads of *env *variants in tissues with and without apparent lymphoma (analogous to Fig. 5D). D) Viral (cDNA) loads of FeLV-A/Glasgow-1 in tissues with and without apparent lymphoma. Viral loads were normalized to RPS7 cDNA copy numbers determined by TaqMan real-time PCR, as described [27]. Viral loads were tested for statistically significant differences using the Mann-Whitney U-test (p_MWU _as indicated). Click here for file

## References

[B1] Jarrett WF Crawford EM Martin WM Davie F A virus-like particle associated with leukaemia (lymphosarcoma) Nature 1964 202 567 568 10.1038/202567a0 14195054

[B2] Pedersen NC Ho EW Brown ML Yamamoto JK Isolation of a T-lymphotropic virus from domestic cats with an immunodeficiency-like syndrome Science 1987 235 790 793 10.1126/science.3643650 3643650

[B3] Yamamoto JK Sparger E Ho EW Andersen PR OConner TP Mandell CP Lowenstinde L Munn R Pedersen NC Pathogenesis of experimentally induced feline immunodeficiency virus infection in cats Am J Vet Res 1988 49 1246 1258 2459996

[B4] Hoover EA Mullins JI Feline leukemia virus infection and diseases J Am Vet Med Assoc. 1991 199 10 1287 1297 1666070

[B5] Flynn JN Dunham SP Watson V Jarrett O Longitudinal analysis of feline leukemia virus-specific cytotoxic T lymphocytes: correlation with recovery from infection J Virol 2002 76 5 2306 2315 10.1128/jvi.76.5.2306-2315.2002 11836409 PMC135947

[B6] Hayes KA Rojko JL Tarr MJ Polas PJ Olsen RG Mathes LE Atypical localised viral expression in a cat with feline leukaemia Vet Rec 1989 124 13 344 346 2541530 10.1136/vr.124.13.344

[B7] Pacitti AM Jarrett O Hay D Transmission of feline leukaemia virus in the milk of a non-viraemic cat Vet Rec 1986 118 14 381 384 3012849 10.1136/vr.118.14.381

[B8] Madewell BR Jarrett O Recovery of feline leukaemia virus from non-viraemic cats Vet Rec 1983 112 15 339 342 6304986 10.1136/vr.112.15.339

[B9] Rojko JL Hoover EA Quackenbush SL Olsen RG Reactivation of latent feline leukaemia virus infection Nature 1982 298 5872 385 388 10.1038/298385a0 6283387

[B10] Post JE Warren L Hardy WD Jr, Essex M, McClelland AJ Reactivation of latent feline leukemia virus Feline Leukemia Virus 1980 New York: Elsevier North Holland Inc 151 155

[B11] Pedersen NC Meric SM Johnson L Plucker S Theiler GH The clinical significance of latent feline leukemia virus infection in cats Feline Pratice 1984 14 32 48

[B12] Pacitti AM Jarrett O Duration of the latent state in feline leukaemia virus infections Vet Rec 1985 117 18 472 474 3000061 10.1136/vr.117.18.472-a

[B13] Hofmann-Lehmann R Huder JB Gruber S Boretti F Sigrist B Lutz H Feline leukaemia provirus load during the course of experimental infection and in naturally infected cats J Gen Virol 2001 82 Pt 7 1589 1596 11413369 10.1099/0022-1317-82-7-1589

[B14] Torres AN Mathiason CK Hoover EA Re-examination of feline leukemia virus: host relationships using real-time PCR Virology 2005 332 1 272 283 10.1016/j.virol.2004.10.050 15661159

[B15] Tandon R Cattori V Gomes-Keller MA Meli ML Golder MC Lutz H Hofmann-Lehmann R Quantitation of feline leukaemia virus viral and proviral loads by TaqMan real-time polymerase chain reaction J Virol Methods 2005 130 1-2 124 132 10.1016/j.jviromet.2005.06.017 16054243

[B16] Hofmann-Lehmann R Cattori V Tandon R Boretti FS Meli ML Riond B Lutz H How molecular methods change our views of FeLV infection and vaccination Vet Immunol Immunopathol 2008 123 1-2 119 123 10.1016/j.vetimm.2008.01.017 18295346

[B17] Hofmann-Lehmann R Cattori V Tandon R Boretti FS Meli ML Riond B Pepin AC Willi B Ossent P Lutz H Vaccination against the feline leukaemia virus: outcome and response categories and long-term follow-up Vaccine 2007 25 30 5531 5539 10.1016/j.vaccine.2006.12.022 17240486

[B18] Hofmann-Lehmann R Tandon R Boretti FS Meli ML Willi B Cattori V Gomes-Keller MA Ossent P Golder MC Flynn JN Lutz H Reassessment of feline leukaemia virus (FeLV) vaccines with novel sensitive molecular assays Vaccine 2006 24 8 1087 1094 10.1016/j.vaccine.2005.09.010 16198454

[B19] Lehmann R Franchini M Aubert A Wolfensberger C Cronier J Lutz H Vaccination of cats experimentally infected with feline immunodeficiency virus, using a recombinant feline leukemia virus vaccine J Am Vet Med Assoc 1991 199 10 1446 1452 1666101

[B20] Hofmann-Lehmann R Holznagel E Ossent P Lutz H Parameters of disease progression in long-term experimental feline retrovirus (feline immunodeficiency virus and feline leukemia virus) infections: Hematology, clinical chemistry and lymphocyte subsets Clin Diagn Lab Immunol 1997 4 1 33 42 9008278 10.1128/cdli.4.1.33-42.1997 PMC170472

[B21] Lutz H Pedersen NC Durbin R Theilen GH Monoclonal antibodies to three epitopic regions of feline leukemia virus p27 and their use in enzyme-linked immunosorbent assay of p27 J Immunol Methods 1983 56 2 209 220 10.1016/0022-1759(83)90413-1 6186744

[B22] Calzolari M Young E Cox D Davis D Lutz H Serological diagnosis of feline immunodeficiency virus infection using recombinant transmembrane glycoprotein Vet Immunol Immunopathol 1995 46 1-2 83 92 10.1016/0165-2427(94)07008-U 7618262

[B23] Hofmann-Lehmann R Holznagel E Aubert A Ossent P Reinacher M Lutz H Recombinant FeLV vaccine: long-term protection and effect on course and outcome of FIV infection Vet Immunol Immunopathol 1995 46 1-2 127 137 10.1016/0165-2427(94)07012-V 7618252 PMC7119625

[B24] Reinacher M Theilen G Frequency and significance of feline leukemia virus infection in necropsied cats Am J Vet Res 1987 48 6 939 945 3037951

[B25] Kipar A Kremendahl J Grant CK von Bothmer I Reinacher M Expression of viral proteins in feline leukemia virus-associated enteritis Vet Pathol 2000 37 2 129 136 10.1354/vp.37-2-129 10714641

[B26] Monteith CE Chelack BJ Davis WC Haines DM Identification of monoclonal antibodies for immunohistochemical staining of feline B lymphocytes in frozen and formalin-fixed paraffin-embedded tissues Can J Vet Res 1996 60 3 193 198 8809382 PMC1263832

[B27] Kessler Y Helfer-Hungerbuehler AK Cattori V Meli ML Zellweger B Ossent P Riond B Reusch CE Lutz H Hofmann-Lehmann R Quantitative TaqMan(R) real-time PCR assays for gene expression normalisation in feline tissues BMC Mol Biol 2009 10 1 106 10.1186/1471-2199-10-106 20003366 PMC2803789

[B28] Cattori V Tandon R Riond B Pepin AC Lutz H Hofmann-Lehmann R The kinetics of feline leukaemia virus shedding in experimentally infected cats are associated with infection outcome Vet Microbiol 2009 133 3 292 296 10.1016/j.vetmic.2008.07.001 18774240

[B29] Meli ML Cattori V Martinez F Lopez G Vargas A Simon MA Zorrilla I Munoz A Palomares F Lopez-Bao JV Pastor J Tandon R Willi B Hofmann-Lehmann R Lutz H Feline leukemia virus and other pathogens as important threats to the survival of the critically endangered Iberian lynx (Lynx pardinus) PLoS ONE 2009 4 3 e4744 10.1371/journal.pone.0004744 19270739 PMC2649436

[B30] Cattori V Tandon R Pepin A Lutz H Hofmann-Lehman R Rapid detection of feline leukemia virus provirus integration into feline genomic DNA Mol Cell Probes 2006 20 3-4 172 181 10.1016/j.mcp.2005.11.007 16488115

[B31] Leutenegger CM Mislin CN Sigrist B Ehrengruber MU Hofmann-Lehmann R Lutz H Quantitative real-time PCR for the measurement of feline cytokine mRNA Vet Immunol Immunopathol 1999 71 291 305 10.1016/S0165-2427(99)00100-2 10587308 PMC7119904

[B32] Leutenegger CM Klein D Hofmann-Lehmann R Mislin C Hummel U Boni J Boretti F Guenzburg WH Lutz H Rapid feline immunodeficiency virus provirus quantitation by polymerase chain reaction using the TaqMan fluorogenic real-time detection system J Virol Methods. 1999 78 1-2 105 116 10.1016/s0166-0934(98)00166-9 10204701

[B33] Cattori V Hofmann-Lehmann R Absolute quantitation of feline leukemia virus proviral DNA and viral RNA loads by TaqMan real-time PCR and RT-PCR Methods Mol Biol 2008 429 73 87 full_text 18695960 10.1007/978-1-60327-040-3_6

[B34] Allenspach K Amacker M Leutenegger CM Hottiger M Hofmann-Lehmann R Hübscher U Pistello M Lutz H Quantification of proviral FIV DNA using competitive PCR Schweiz Arch Tierheilkd 1996 138 87 92 8720733

[B35] Hofmann-Lehmann R Williams AL Swenerton RK Li PL Rasmussen RA Chenine AL McClure HM Ruprecht RM Quantitation of simian cytokine and beta-chemokine mRNAs, using real-time reverse transcriptase-polymerase chain reaction: variations in expression during chronic primate lentivirus infection AIDS Res Hum Retroviruses 2002 18 9 627 639 10.1089/088922202760019329 12079558

[B36] Tandon R Cattori V Willi B Lutz H Hofmann-Lehmann R Quantification of endogenous and exogenous feline leukemia virus sequences by real-time PCR assays Vet Immunol Immunopathol 2008 123 1-2 129 133 10.1016/j.vetimm.2008.01.027 18295344

[B37] Sheets RL Pandey R Jen WC Roy-Burman P Recombinant feline leukemia virus genes detected in naturally occurring feline lymphosarcomas J Virol 1993 67 6 3118 3125 8388492 10.1128/jvi.67.6.3118-3125.1993 PMC237649

[B38] Mathes LE Pandey R Chakrabarti R Hofman FM Hayes KA Stromberg P Roy-Burman P Pathogenicity of a subgroup C feline leukemia virus (FeLV) is augmented when administered in association with certain FeLV recombinants Virology 1994 198 185 195 10.1006/viro.1994.1021 8259654

[B39] Büttner A Vergleichende Untersuchungen über Translation und Transkription von Strukturproteinen des Felinen Leukämievirus nach experimenteller Infektion Inaugural dissertation 2004 Giessen, Germany: University of Giessen

[B40] Donahue PR Hoover EA Beltz GA Riedel N Hirsch VM Overbaugh J Mullins JI Strong sequence conservation among horizontally transmissible, minimally pathogenic feline leukemia viruses J Virol 1988 62 722 731 2828667 10.1128/jvi.62.3.722-731.1988 PMC253625

[B41] Nishigaki K Okuda M Endo Y Watari T Tsujimoto H Hasegawa A Structure and function of the long terminal repeats of feline leukemia viruses derived from naturally occurring acute myeloid leukemias in cats J Virol 1997 71 12 9823 9827 9371654 10.1128/jvi.71.12.9823-9827.1997 PMC230298

[B42] Kumar S Nei M Dudley J Tamura K MEGA: a biologist-centric software for evolutionary analysis of DNA and protein sequences Brief Bioinform 2008 9 4 299 306 10.1093/bib/bbn017 18417537 PMC2562624

[B43] Thompson JD Higgins DG Gibson TJ CLUSTAL W: improving the sensitivity of progressive multiple sequence alignment through sequence weighting, position-specific gap penalties and weight matrix choice Nucleic Acids Res 1994 22 22 4673 4680 10.1093/nar/22.22.4673 7984417 PMC308517

[B44] Felsenstein J Confidence Limits on Phylogenies: An Approach Using the Bootstrap Evolution 1985 39 4 783 791 10.2307/2408678 28561359

[B45] Nei M Kumar S Molecular Evolution and Phylogenetics 2000 New York: Oxford University Press

[B46] Werner JA Woo JC Vernau W Graham PS Grahn RA Lyons LA Moore PF Characterization of feline immunoglobulin heavy chain variable region genes for the molecular diagnosis of B-cell neoplasia Vet Pathol 2005 42 5 596 607 10.1354/vp.42-5-596 16145206

[B47] Moore PF Woo JC Vernau W Kosten S Graham PS Characterization of feline T cell receptor gamma (TCRG) variable region genes for the molecular diagnosis of feline intestinal T cell lymphoma Vet Immunol Immunopathol 2005 106 3-4 167 178 10.1016/j.vetimm.2005.02.014 15963816

[B48] Henrich M Hecht W Weiss AT Reinacher M A new subgroup of immunoglobulin heavy chain variable region genes for the assessment of clonality in feline B-cell lymphomas Vet Immunol Immunopathol 2009 130 1-2 59 69 10.1016/j.vetimm.2009.01.006 19243841

[B49] Greiner M Serodiagnostische Tests Evaluierung und Interpretation in der Veterinärmedizin und anderen Fachgebieten Springer, Berlin, Germany 2003

[B50] Landis JR Koch GG The measurement of observer agreement for categorical data Biometrics 1977 33 1 159 174 10.2307/2529310 843571

[B51] Valli VE Jacobs RM Parodi AL Vernau W Moore PF World Health Organization International Histological Classification of Tumors of Domestic Animals: Histological Classification of Hematopoietic Tumors of Domestic Animals 2002 VIII Armed Force Institute of Pathology, American Registry of Pathology

[B52] Rey MA Prasad R Tailor CS The C domain in the surface envelope glycoprotein of subgroup C feline leukemia virus is a second receptor-binding domain Virology 2008 370 2 273 284 10.1016/j.virol.2007.09.011 17945326

[B53] Stewart MA Warnock M Wheeler A Wilkie N Mullins JI Onions DE Neil JC Nucleotide sequences of a feline leukemia virus subgroup A envelope gene and long terminal repeat and evidence for the recombinational origin of subgroup B viruses J Virol 1986 58 825 834 3009890 10.1128/jvi.58.3.825-834.1986 PMC252989

[B54] Shelton GH Grant CK Cotter SM Gardner MB Hardy WD Jr DiGiacomo RF Feline immunodeficiency virus and feline leukemia virus infections and their relationships to lymphoid malignancies in cats: a retrospective study (1968-1988) J Acquir Immune Defic Syndr 1990 3 623 630 2159993

[B55] Chandhasin C Coan PN Pandrea I Grant CK Lobelle-Rich PA Puetter A Levy LS Unique long terminal repeat and surface glycoprotein gene sequences of feline leukemia virus as determinants of disease outcome J Virol 2005 79 9 5278 5287 10.1128/JVI.79.9.5278-5287.2005 15827142 PMC1082761

[B56] Jarrett O Pathogenicity of feline leukemia virus is commonly associated with variant viruses Leukemia 1992 6 Suppl 3 153S 154S 1318467

[B57] Neil JC Fulton R Rigby M Stewart M Feline leukaemia virus: generation of pathogenic and oncogenic variants Curr Top Microbiol Immunol 1991 171 67 93 1667630 10.1007/978-3-642-76524-7_4

[B58] Shelton GH McKim KD Cooley PL Dice PF Russell RG Grant CK Feline leukemia virus and feline immunodeficiency virus infections in a cat with lymphoma J Am Vet Med Assoc 1989 194 2 249 252 2537274

[B59] Callanan JJ Jones BA Irvine J Willett BJ McCandlish IA Jarrett O Histologic classification and immunophenotype of lymphosarcomas in cats with naturally and experimentally acquired feline immunodeficiency virus infections Vet Pathol 1996 33 3 264 272 8740699 10.1177/030098589603300302

[B60] Poli A Abramo F Baldinotti F Pistello M Da Prato L Bendinelli M Malignant lymphoma associated with experimentally induced feline immunodeficiency virus infection J Comp Pathol 1994 110 319 328 10.1016/S0021-9975(08)80309-X 7914523

[B61] Gabor LJ Love DN Malik R Canfield PJ Feline immunodeficiency virus status of Australian cats with lymphosarcoma Aust Vet J 2001 79 8 540 545 10.1111/j.1751-0813.2001.tb10742.x 11599813

[B62] Beatty JA Callanan JJ Terry A Jarrett O Neil JC Molecular and immunophenotypical characterization of a feline immunodeficiency virus (FIV)-associated lymphoma: a direct role for FIV in B-lymphocyte transformation? J Virol 1998 72 1 767 771 9420284 10.1128/jvi.72.1.767-771.1998 PMC109433

[B63] Feichtinger H Putkonen P Parravicini C Li SL Kaaya EE Bottiger D Biberfeld G Biberfeld P Malignant lymphomas in cynomolgus monkeys infected with simian immunodeficiency virus Am J Pathol 1990 137 6 1311 1315 1701962 PMC1877739

[B64] Levine AM Acquired immunodeficiency syndrome-related lymphoma Blood 1992 80 1 8 20 1319239

[B65] Jackson ML Wood SL Misra V Haines DM Immunohistochemical identification of B and T lymphocytes in formalin-fixed, paraffin-embedded feline lymphosarcomas: relation to feline leukemia virus status, tumor site, and patient age Can J Vet Res 1996 60 3 199 204 8809383 PMC1263833

[B66] Phipps AJ Chen H Hayes KA Roy-Burman P Mathes LE Differential pathogenicity of two feline leukemia virus subgroup A molecular clones, pFRA and pF6A J Virol 2000 74 13 5796 5801 10.1128/JVI.74.13.5796-5801.2000 10846058 PMC112073

[B67] Beatty JA Lawrence CE Callanan JJ Grant CK Gault EA Neil JC Jarrett O Feline immunodeficiency virus (FIV)-associated lymphoma: a potential role for immune dysfunction in tumourigenesis Vet Immunol Immunopathol 1998 65 2-4 309 322 10.1016/S0165-2427(98)00164-0 9839882

[B68] Hamilton-Dutoit SJ Pallesen G Franzmann MB Karkov J Black F Skinhoj P Pedersen C AIDS-related lymphoma. Histopathology, immunophenotype, and association with Epstein-Barr virus as demonstrated by in situ nucleic acid hybridization Am J Pathol 1991 138 1 149 163 1846263 PMC1886047

[B69] Overbaugh J Bangham CR Selection forces and constraints on retroviral sequence variation Science 2001 292 5519 1106 1109 10.1126/science.1059128 11352065

[B70] Jarrett O Russell PH Differential growth and transmission in cats of feline leukaemia viruses of subgroups A and B Int J Cancer 1978 21 466 472 10.1002/ijc.2910210411 208983

[B71] Overbaugh J Riedel N Hoover EA Mullins JI Transduction of endogenous envelope genes by feline leukaemia virus in vitro Nature 1988 332 731 734 10.1038/332731a0 2895894

[B72] Rigby MA Rojko JL Stewart MA Kociba GJ Cheney CM Rezanka LJ Mathes LE Hartke JR Jarrett O Neil JC Partial dissociation of subgroup C phenotype and in vivo behaviour in feline leukaemia viruses with chimeric envelope genes J Gen Virol 1992 73 2839 2847 10.1099/0022-1317-73-11-2839 1331290

[B73] Sarangi A Bupp K Roth MJ Identification of a retroviral receptor used by an envelope protein derived by peptide library screening Proc Natl Acad Sci USA 2007 104 26 11032 11037 10.1073/pnas.0704182104 17581869 PMC1904118

[B74] Mazari PM Linder-Basso D Sarangi A Chang Y Roth MJ Single-round selection yields a unique retroviral envelope utilizing GPR172A as its host receptor Proc Natl Acad Sci USA 2009 106 14 5848 5853 10.1073/pnas.0809741106 19307586 PMC2667028

[B75] Bupp K Sarangi A Roth MJ Probing sequence variation in the receptor-targeting domain of feline leukemia virus envelope proteins with peptide display libraries J Virol 2005 79 3 1463 1469 10.1128/JVI.79.3.1463-1469.2005 15650172 PMC544108

[B76] Chandhasin C Coan PN Levy LS Subtle mutational changes in the SU protein of a natural feline leukemia virus subgroup A isolate alter disease spectrum J Virol 2005 79 3 1351 1360 10.1128/JVI.79.3.1351-1360.2005 15650161 PMC544135

[B77] Jackson ML Haines DM Misra V Sequence analysis of the putative viral enhancer in tissues from 33 cats with various feline leukemia virus-related diseases Vet Microbiol 1996 53 3-4 213 225 10.1016/S0378-1135(96)01228-X 9008333

[B78] Fulton R Plumb M Shield L Neil JC Structural diversity and nuclear protein binding sites in the long terminal repeats of feline leukemia virus J Virol 1990 64 1675 1682 2157050 10.1128/jvi.64.4.1675-1682.1990 PMC249304

[B79] Matsumoto Y Momoi Y Watari T Goitsuka R Tsujimoto H Hasegawa A Detection of enhancer repeats in the long terminal repeats of feline leukemia viruses from cats with spontaneous neoplastic and nonneoplastic diseases Virology 1992 189 745 749 10.1016/0042-6822(92)90598-J 1322598

[B80] Miura T Shibuya M Tsujimoto H Fukasawa M Hayami M Molecular cloning of a feline leukemia provirus integrated adjacent to the c-myc gene in a feline T-cell leukemia cell line and the unique structure of its long terminal repeat Virology 1989 169 458 461 10.1016/0042-6822(89)90172-4 2539700

[B81] Levy LS Advances in understanding molecular determinants in FeLV pathology Vet Immunol Immunopathol 2008 123 1-2 14 22 10.1016/j.vetimm.2008.01.008 18289704 PMC2413067

[B82] Francis DP Essex M Hardy WD Jr Excretion of feline leukaemia virus by naturally infected pet cats Nature 1977 269 252 254 10.1038/269252a0 201852

[B83] Elder JH Mullins JI Nucleotide sequence of the envelope gene of Gardner-Arnstein feline leukemia virus B reveals unique sequence homologies with a murine mink cell focus-forming virus J Virol 1983 46 871 880 6304347 10.1128/jvi.46.3.871-880.1983 PMC256562

