# Supplementary figures and images for: Dominance of highly divergent feline leukemia virus A progeny variants in a cat with recurrent viremia and fatal lymphoma
Source: Retrovirology. 2010 Feb 19;7:14. doi: 10.1186/1742-4690-7-14 (PMC2837606; doi:10.1186/1742-4690-7-14)

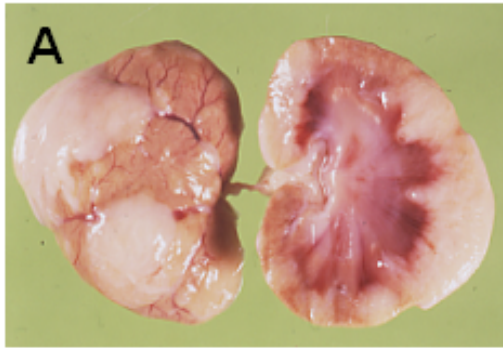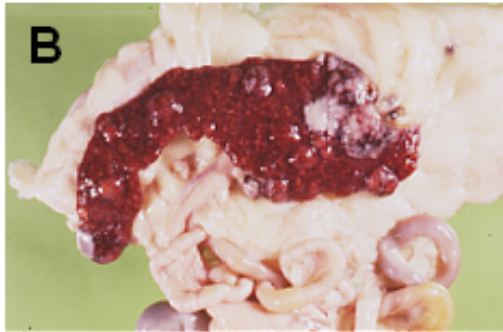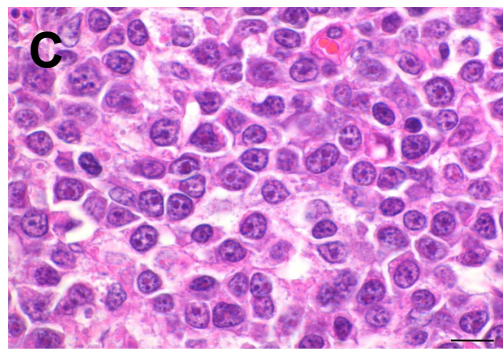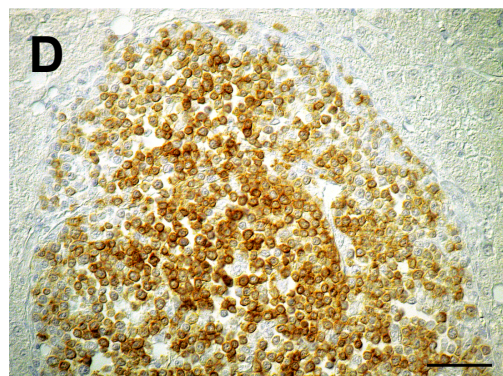

Supplement: Additional file 1 — Multicentric lymphoma. Lymphoma detected upon necropsy. A) Kidney. B) Spleen. C) Histology of sternal lymph node: diffuse proliferation of mainly medium sized lymphatic cells with round nuclei, coarsely stippled chromatin and one to multiple medium sized nucleoli. Hematoxylin and Eosin, bar = 10 μm. D) Liver: positive immunohistochemical labeling of periportal infiltrating neoplastic cells for CD45R. Avidin-biotin complex method; Papanicolaou's hematoxylin counterstain, bar = 50 μm. [file 1742-4690-7-14-S1.PDF]

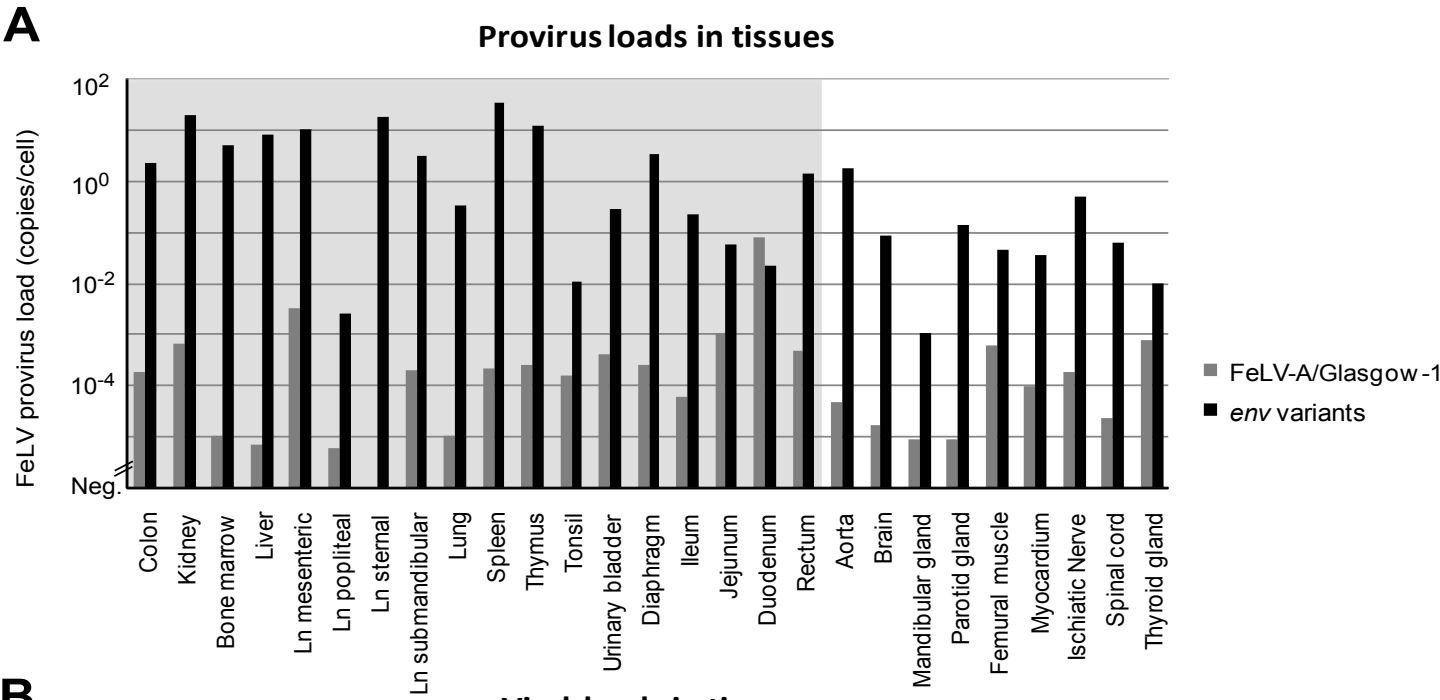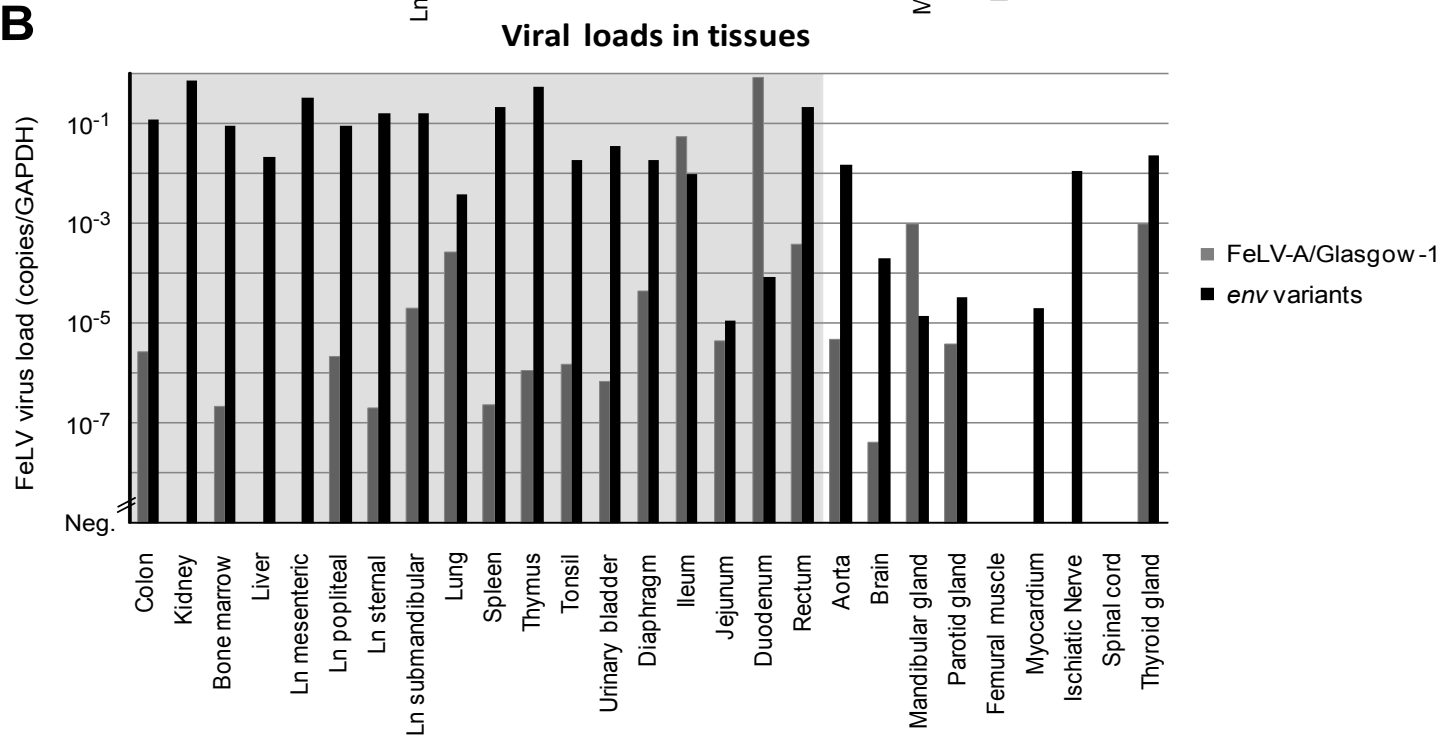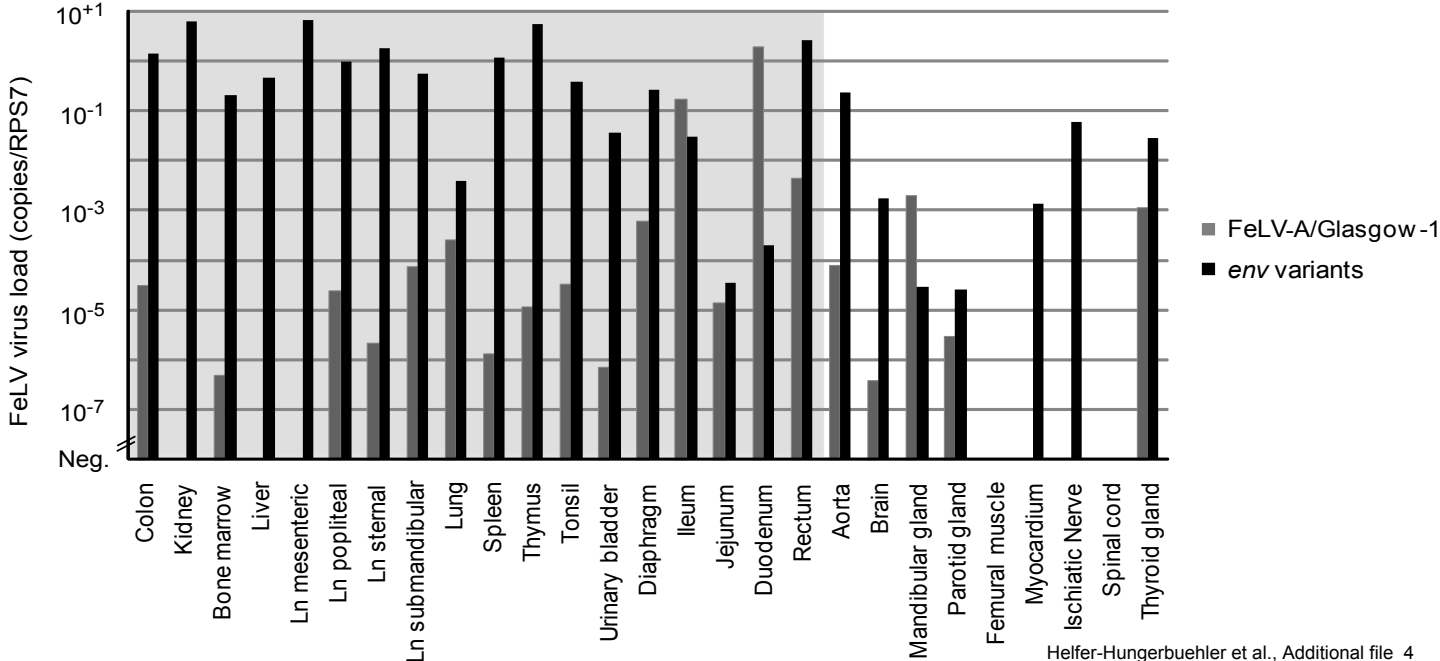

Supplement: Additional file 4 — FeLV-A/Glasgow-1 and env variant provirus and viral loads in the tissues from cat #261. A) Provirus loads of FeLV-A/Glasgow-1 and the env variants. B) Viral (cDNA) loads of FeLV-A/Glasgow-1 and env variants. Viral tissue loads were normalized to GAPDH (top) and to RPS7 cDNA copy numbers (bottom). Tissues with apparent lymphoma are indicated by shaded areas. [file 1742-4690-7-14-S4.PDF]

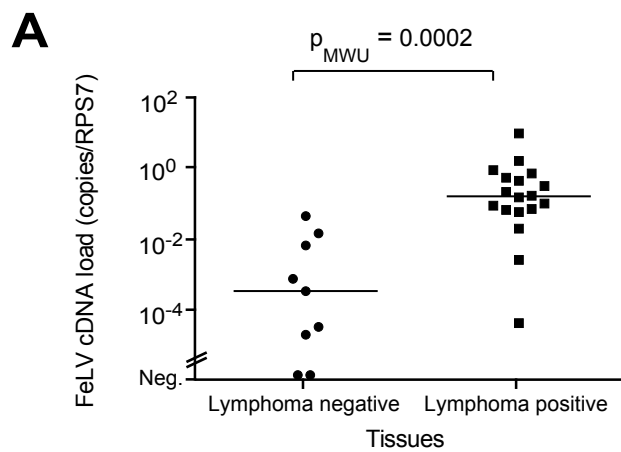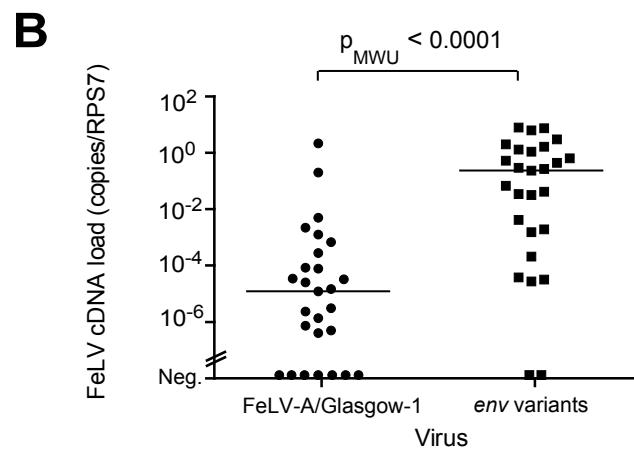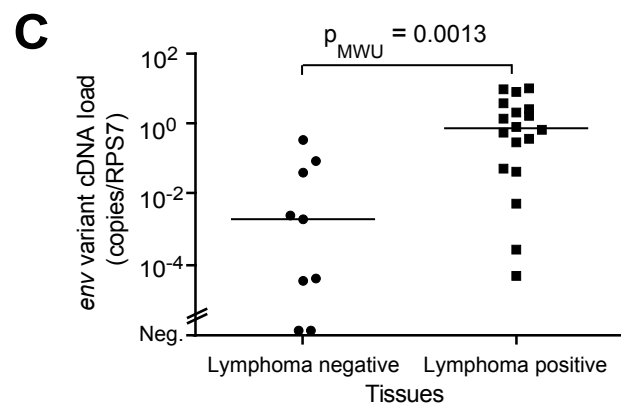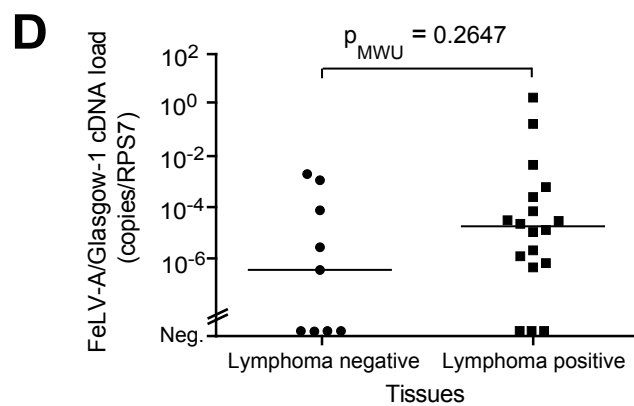

Supplement: Additional file 5 — FeLV viral loads in tissues from cat #261 normalized to RPS7. A) Total FeLV viral (cDNA) loads (U3 region PCR) in tissues with and without apparent lymphoma (analogous to Fig. 3C). B) Viral (cDNA) loads of FeLV-A/Glasgow-1 and env variants (analogous to Fig. 5C). C) Viral (cDNA) loads of env variants in tissues with and without apparent lymphoma (analogous to Fig. 5D). D) Viral (cDNA) loads of FeLV-A/Glasgow-1 in tissues with and without apparent lymphoma. Viral loads were normalized to RPS7 cDNA copy numbers determined by TaqMan real-time PCR, as described [27]. Viral loads were tested for statistically significant differences using the Mann-Whitney U-test (pMWU as indicated). [file 1742-4690-7-14-S5.PDF]
