# Supplementary material for: Dominance of highly divergent feline leukemia virus A progeny variants in a cat with recurrent viremia and fatal lymphoma
Source: Retrovirology. 2010 Feb 19;7:14. doi: 10.1186/1742-4690-7-14 (PMC2837606; doi:10.1186/1742-4690-7-14)
Supplement: Additional file 2 — Amino acid alignment of FeLV env sequences. Amino acid alignment of the env coding region from three FeLV subtypes (FeLV-A/Glasgow-I [GenBank: M12500], FeLV-B/Gardner-Arnstein [GenBank: K01209] and FeLV-C/Sarma [GenBank: M14331]) and the three env variants (KI261-I, KI261-II and SP261-III). The start of the SU region, the transmembrane domain (TM), the variable regions VRA, VRB and VRC, the PRR and the C2 disulfide-bonded loop (S-S) are labeled (according to [52]). Circles and stars represent amino acid sequences containing Asn-X-Ser/Thr, which indicate possible sites of N-glycosylation, as previously described [53,83]. Potential N-linked glycosylation sites that are conserved in FeLV-A/Glasgow-1 and all env variants are represented by filled circles. New potential N-linked glycosylation sites in the env variants that were not present in the challenge strain FeLV-A/Glasgow-1 are labeled with filled stars; those that were present in FeLV-A/Glasgow-1 but lost in the env variants are marked with empty stars. Dots represent identical residues, and dashes represent spaces, which were introduced for proper alignment. [file 1742-4690-7-14-S2.PDF]

Alignment of the three FeLV subtypes with the *env* variants from cat #261

```
                           Start of SU
FeLV-A      1   MESPTHPKPSKDKTLSWNLAFLVGILFTIDIGMANPSPHQIYNVTWVITNVQTNTQANAT  60
FeLV-B      1   ......................V.................V.....T...LV.G.K....  60
FeLV-C      1   ..............FP......V....Q..M.........V.............SR....  60
KI261-I     1   ...S............GY......F.C.V...............................  60
KI261-II    1   ...S.............Y......................V.....I...I....R....  60
SP261-III   1   ...S............GY......F............................GA...T.  60

                                                   VRA
FeLV-A     61   SMLGTLTDAYPTLHVDLCDLVGDTWEPIVLNPTNVKHGARYSSSKYGCKTTDRKKQQQTY 120
FeLV-B     61   .........F..MYF....II.N..N.SDQE.----------FPG...DQPM.RW..RNT 110
FeLV-C     61   .............Y..............APD.---RSW......TH.............. 117
KI261-I    61   ...............................SH......L.YTP......PE.R....A. 120
KI261-II   61   .........F............N....K...SHY.R...L.PT.....L.E.......A. 120
SP261-III  61   ................................HST....L.YTP......PE.R....A. 120

                      VRC
FeLV-A    121   PFYVCPGHAPSLGPKGTHCGGAQDGFCAAWGCETTGEAWWKPTSSWDYITVKRGSSQ--- 177
FeLV-B    111   .........NRK-----Q...P......V........TY.R...........K.VT.GIY 165
FeLV-C    118   ...........M.....Y.....................................N.--- 174
KI261-I   121   ..........LM.....Y..........S.......D..............N..D.P--- 177
KI261-II  121   .........................Y..........D...N..........RK.I..--- 177
SP261-III 121   ..........LM.....Y.......Y..........D..............T.....--- 177

                        VRB
FeLV-A    177   ------------------DNS---CEGKCNPLVLQFTQKGRQASWDGPKMWGLRLYRTGY 216
FeLV-B    166   QCSGGGWCGPCYDKAVHSSTTGASEG.R....I.........T......S.......S.. 225
FeLV-C    174   ------------------...---.K....................R..........S.. 213
KI261-I   177   ------------------NSN---.T.................................. 216
KI261-II  177   ------------------NSN-CGGGK.............K................... 218
SP261-III 177   ------------------...DEA.NT....................R.A.......... 219

                                                 PRR
FeLV-A    217   DPIALFTVSRQVSTITPPQAMGPNLVLPDQKPPSRQSQTGSKVATQRPQTN--------- 267
FeLV-B    226   ......S.....M.........................IE.R.TPHHS.G.GGTPGITLV 285
FeLV-C    214   ......S.....M..........................K...T.....IT--------- 264
KI261-I   217   ...........................LN......---.............--------- 264
KI261-II  219   ..V...S.....................N......---....A...E.L..--------- 266
SP261-III 220   ............................N.....Q---.............--------- 267

FeLV-A    267   --ESAPRSVAPTTMGPKRIGTGDRLINLVQGTYLALNATDPNKTKDCWLCLVSRPPYYEG 325
FeLV-B    286   NASI..L.TPV.PAS...........................R................. 345
FeLV-C    264   --S.T.....SA................................................ 322
KI261-I   264   --.....T.T.P.LN..W.....................N.................... 322
KI261-II  266   --......IP.P.IN.NW.....................N.................... 324
SP261-III 267   --...L...T.P.IN..W.......................................... 325

                                         C2 loop
FeLV-A    326   IAILGNYSNQTNPPPSCLSTPQHKLTISEVSGQGMCIGTVPKTHQALCNKTQQGHTGAHY 385
FeLV-B    346   ...................I..............L..............E.......... 405
FeLV-C    323   ..V...............................L.............K...K..K.T.. 382
KI261-I   323   ...........S.L.....H.P.R........R.T..............Q..P..E.G.. 382
KI261-II  325   .......T...Q..L...................I..............E.....N.T.. 384
SP261-III 326   V..........S................................R.........YN.TR. 385
```

```
                   C2 loop
FeLV-A      386 LAAPNGTYWACNTGLTPCISMAVLNWTSDFCVLIELWPRVTYHQPEYVYTHFAKAVRFRR 445
FeLV-B      406 .......................................................A.... 465
FeLV-C      383 ................................................I...D....... 442
KI261-I     383 .I...........................................S.......G...... 442
KI261-II    385 .V...........................................S..I.S......... 444
SP261-III   386 .V...........................................S..M........... 445

                Start of TM
FeLV-A      446 EPISLTVALMLGGLTVGGIAAGVGTGTKALLETAQFRQLQMAMHTDIQALEESISALEKS 505
FeLV-B      466 ..............................I............................. 525
FeLV-C      443 ........................................I................... 502
KI261-I     443 ..V........................A.........................V...... 502
KI261-II    445 D.V...............V........A................................ 504
SP261-III   446 ...........................T................................ 505

FeLV-A      506 LTSLSEVVLQNRRGLDILFLQEGGLCAALKEECCFYADHTGLVRDNMAKLRERLKQRQQL 565
FeLV-B      526 ............................................................ 585
FeLV-C      503 ............................................................ 562
KI261-I     503 ..............................................S.......R..... 562
KI261-II    505 ..............................................S.......R..... 564
SP261-III   506 ..............................................S.......R..... 565

                            ☆
FeLV-A      566 FDSQQGWFEGWFNKSPWFTTLISSIMGPLLILLLILLFGPCILNRLVQFVKDRISVVQAL 625
FeLV-B      586 ............................................................ 645
FeLV-C      563 .....................................L...................... 622
KI261-I     563 ............S............T.................................. 622
KI261-II    565 .........................T.................................. 624
SP261-III   566 ...ER....................T.................................. 625

FeLV-A      626 ILTQQYQQIKQYDPDRP 642
FeLV-B      646 ................. 662
FeLV-C      623 .........Q...S... 639
KI261-I     623 .........R....... 639
KI261-II    625 ........VR....... 641
SP261-III   626 .........R....... 642
```
