# Supplementary material for: Dominance of highly divergent feline leukemia virus A progeny variants in a cat with recurrent viremia and fatal lymphoma
Source: Retrovirology. 2010 Feb 19;7:14. doi: 10.1186/1742-4690-7-14 (PMC2837606; doi:10.1186/1742-4690-7-14)
Supplement: Additional file 3 — Comparison of nucleotide sequences of the FeLV U3 region. Nucleotide sequence comparison of the U3 region from FeLV-A/Glasgow-1 [GenBank: M12500] and the progeny virus variants retrieved from cat #261. Sequences found in multiple clones were depicted once. KI261-I was found in clones from the kidney. SP261-III, pKH11.8 and pKH11.9 were found in clones from the spleen. All other listed sequences were found in at least two different tissues, including the kidney, liver, spleen, and bone marrow. Enhancer elements and their corresponding nucleotide sequences are marked in the reference strain FeLV-A/Glasgow-1 [GenBank: M12500]. Nucleotides differing from the originally inoculated strain are indicated. Primer sequences for PCR [41] were located at positions -415 to -392 and 9 to 32 (not included in the figure). Previously described mutations are indicated by numbers in brackets: (1) Jackson et al., 1996; (2) Nishigaki et al., 1997; (3) Fulton et al., 1990 and (4) Matsumoto et al., 1992. Numbers at the top of the sequence indicate nucleotide positions relative to the presumptive RNA cap site in the FeLV/Glasgow-1 LTR [53]. Grey dashed lines indicate unknown sequences. [file 1742-4690-7-14-S3.PDF]

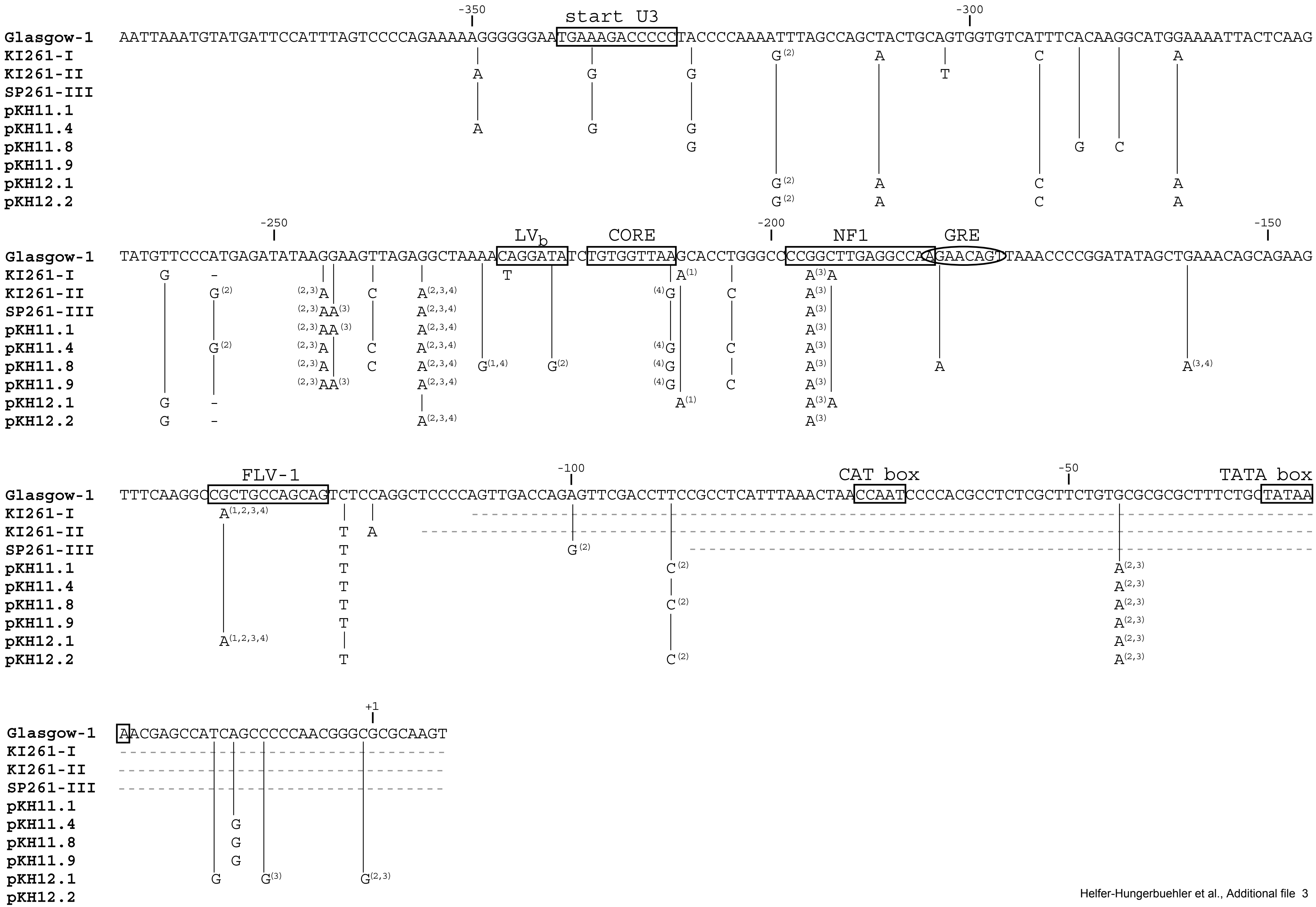

Helfer-Hungerbuehler et al., Additional file 3
